# Cholesterol biosynthesis induced by radiotherapy inhibits cGAS–STING activation and contributes to colorectal cancer treatment resistance

**DOI:** 10.1038/s12276-025-01457-6

**Published:** 2025-05-12

**Authors:** Lijun Zhu, Zhaohui Tang, Wen Jiang, Yuwen Dong, Xiaofei Li, Kai Huang, Tiancong Wu, Lingyan Xu, Wenjie Guo, Yanhong Gu

**Affiliations:** 1 Department of Oncology, The First Affiliated Hospital with Nanjing Medical University, The First Clinical Medical College of Nanjing Medical University, Nanjing Medical University, Nanjing, China; 2 State Key Laboratory of Pharmaceutical Biotechnology, Department of Gastroenterology, Nanjing Drum Tower Hospital, School of Life Science, Nanjing University, Nanjing, China; 3 Department of Gastroenterology and Hepatology, Jinling Hospital, Affiliated Hospital of Medical School, Nanjing University, Nanjing, China

**Keywords:** Pattern recognition receptors, Radiotherapy

## Abstract

Radiotherapy-induced DNA damage can lead to apoptotic cell death and trigger an anti-tumor immune response via the cyclic GMP–AMP synthase–stimulator of interferon genes (cGAS–STING) pathway, which senses cytoplasmic double-stranded DNA. However, radiotherapy resistance poses a significant challenge in treating cancers, including colorectal cancer (CRC). Understanding the mechanisms underlying this resistance is crucial for developing effective therapies. Here we report that radiotherapy enhances cholesterol synthesis, which subsequently inhibits the cGAS–STING pathway, leading to radiotherapy resistance. Mechanistically, 3-hydroxy-3-methylglutaryl-CoA reductase (HMGCR) levels increase rapidly in response to radiation, resulting in increased cholesterol synthesis. This increased cholesterol sequesters STING in the endoplasmic reticulum, hindering its activation and downstream interferon signaling. Elevated HMGCR and cholesterol levels correlate with poor prognosis and reduced response to radiation therapy in patients with CRC. Importantly, pharmacological inactivation of HMGCR significantly enhanced radiotherapy responsiveness in animal models, dependent on cGAS–STING signaling-mediated anti-tumor responses. Our findings reveal that radiotherapy-induced cholesterol inhibits cGAS–STING signaling, facilitating tumor immune escape. Therefore, combining statins with radiotherapy represents a promising therapeutic strategy for treating CRC.

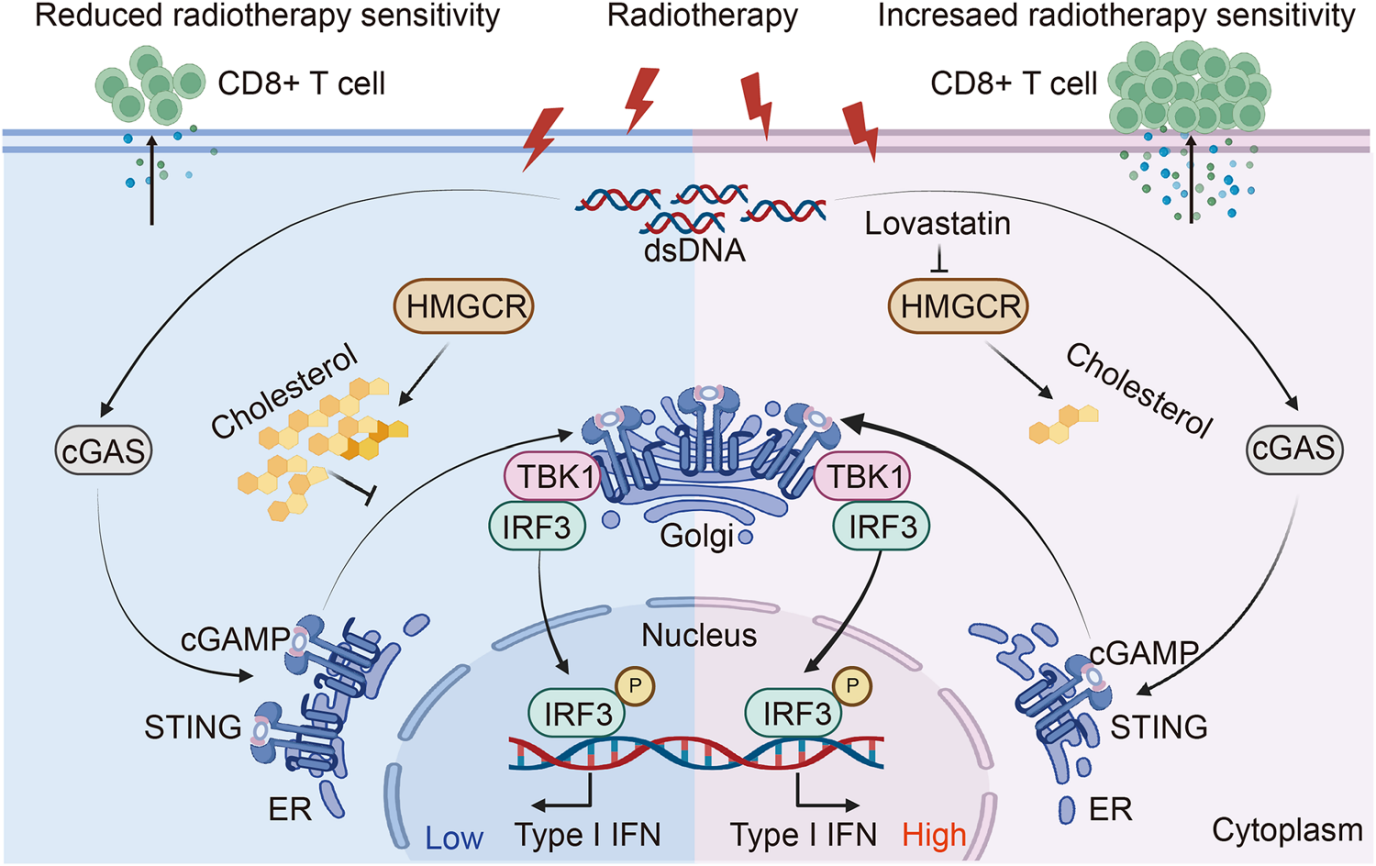

## Radiotherapy boosts cholesterol, leading to cancer resistance

Colorectal cancer (CRC) is a common cancer affecting the digestive system. This study explores how cholesterol levels in tumor cells might affect radiotherapy outcomes. Researchers found that radiation increases cholesterol production in tumor cells, which can reduce the effectiveness of radiotherapy by limiting immune responses. The study used animal models to test whether lowering cholesterol with lovastatin could improve radiotherapy results. The researchers conducted experiments on mice with CRC tumors, combining lovastatin with radiotherapy. They found that this combination enhanced the immune response against tumors by activating a pathway called cGAS–STING, which is crucial for immune activation. This led to increased infiltration of CD8^+^ T cells into the tumors, improving the treatment’s effectiveness. The study concludes that combining lovastatin with radiotherapy could enhance cancer treatment by boosting the body’s immune response.

This summary was initially drafted using artificial intelligence, then revised and fact-checked by the author.

## Introduction

Colorectal cancer (CRC) is the most prevalent malignant tumor of the digestive tract worldwide^1^. Radiotherapy, a mainstay of conventional cancer treatment, plays a critical role in the treatment of CRC^2^. Recent studies have shown that ionizing radiation (IR) not only causes double-stranded DNA (dsDNA) damage, directly leading to tumor cell death, but also activates tumor-intrinsic innate immunity^3,4^. Accumulating evidence indicates that the innate immune system plays a pivotal role in tumor prevention, progression and metastasis, significantly influencing the efficacy of conventional cancer therapies^5^. Although various damage-associated molecular patterns can activate the innate immune system in cancer treatment, the recognition of abnormal DNA by the cyclic GMP–AMP synthase–stimulator of interferon genes (cGAS–STING) pathway is particularly important in innate immune activation^6,7^. Radiotherapy has been reported to induce the accumulation of dsDNA in the cytosol of tumor cells, activating the intrinsic cGAS–STING pathway^8^. This activation subsequently leads to the release of type I interferon (IFN) and other immune cytokines, which are essential for increasing the number and function of CD8^+^ T cells^9^. Therefore, therapeutic strategies targeting the activation of the cGAS–STING pathway have become an important approach to enhance the efficacy of traditional treatments, including radiotherapy^10–13^.

Although radiotherapy can activate the cGAS–STING pathway, the extent of this activation remains limited^14,15^. As a protective mechanism, tumor cells exposed to radiation can initiate negative feedback mechanisms that limit the sustained activity of the cGAS–STING pathway, potentially reducing its effectiveness in the anti-tumor immune response. However, the mechanisms underlying this limited activation are not completely understood. Emerging evidence suggests a link between lipid metabolism and innate immunity^16–18^. Cholesterol, an important metabolite in lipid metabolism, regulates innate immunity^19–22^ and is reported to be a risk factor for the occurrence, progression and resistance of many tumors^23–25^. Furthermore, cholesterol inhibits the transport of STING from the endoplasmic reticulum (ER) to the Golgi apparatus, thereby suppressing the activation of the cGAS–STING pathway and subsequent interferon signaling^26^.

In our study, we found that 3-hydroxy-3-methylglutaryl-CoA reductase (HMGCR) levels rapidly increased in response to radiation stimuli, leading to elevated cholesterol synthesis. This elevated cholesterol level inhibits the activation of the cGAS–STING pathway. Clinical analyses have consistently demonstrated a correlation between high HMGCR and cholesterol levels and diminished responsiveness to radiotherapeutic interventions among patients with CRC. Based on these findings, we used lovastatin, an HMGCR inhibitor, combined with radiotherapy in animal models. The combination therapy induced robust anti-tumor immunity by activating the cGAS–STING pathway, leading to increased infiltration of CD8^+^ T cells into the tumor microenvironment, significantly enhancing the observed therapeutic efficacy. Our study provides a mechanistic foundation for future clinical trials combining radiotherapy with statin lipid-lowering agents for treating CRC and other solid tumors.

## Materials and methods

### Chemicals, reagents and antibodies

C-176 (cat. no. 314054-00-7) and lovastatin (cat. no. 75330-75-5) were obtained from TargetMol. Anti-p-TBK1 (cat. no. 00261) was purchased from Boster Bio. Anti-p-IRF3 (cat. no. 37829S), anti-p-STING (Ser366) (cat. no. 19781S) and anti-STING (cat. no. 13647S) were purchased from Cell Signaling Technology. Anti-TBK1 (cat. no. 28397-1-AP), anti-IRF3 (cat. no. 11312-1-AP), anti-CD8 (cat. no. 66868-1-Ig), anti-H2AX (cat. no. 10856-1-AP), anti-PDI (cat. no. 66422-1-Ig), anti-HMGCR (cat. no. 13533-1-AP), CoraLite488-conjugated goat anti-rabbit IgG (H+L) (cat. no. SA00013-2), CoraLite594-conjugated goat anti-rabbit IgG (H+L) (cat. no. SA00013-4) and Multi-rA CoraLite Plus 647-goat anti-mouse recombinant secondary antibody (H+L) (cat. no. RGAM005) were purchased from Proteintech. Anti-p-cGAS (cat. no. AP1228) and anti-cGAS (cat. no. A8335) were purchased from Abclonal. Anti-GAPDH (cat. no. M20006L) was purchased from Abmart. Anti-PCNA (cat. no. GB11010) was purchased from Servicebio. Anti-Cleaved-Caspase-3 (cat. no. YC0004) was obtained from Immunoway. Anti-IFNβ (cat. no. PA5-102429) was obtained from Thermo Fisher Scientific. Anti-γH2AX (cat. no. 26350), anti-CD4 (cat. no. ab183685) and anti-CD8 (cat. no. ab217344) were purchased from Abcam. Anti-IFN-γ (cat. no. 105995) was purchased from SinoBiological. Total cholesterol assay kit (cat. no. A111-1-1) and lactate dehydrogenase assay kit (cat. no. A020-2-2) were purchased from Nanjing Jiancheng Bioengineering Institute. Cholesterol-boron difluoride dipyrrin complex (BODIPY) (cat. no. HY-125746), water-soluble cholesterol (cat. no. HY-N0322A) and Filipin III (cat. no. HY-N6718) were obtained from MedChemExpress. Hoechst 33342 (cat. no. C1022), propidium iodide (cat. no. ST511) and DAPI (cat. no. C1002) were purchased from Beyotime. Lymphocyte separation medium (cat. no. BL1420) was obtained from Biosharp. Methyl-β-cyclodextrin (MβCD) (cat. no. M102038) was purchased from Aladdin.

### Cell culture

The mouse CRC cell lines CT26 and MC38 and the human CRC cell line HCT116 were purchased from Shanghai Cell Bank of Chinese Academy of Sciences. These cells were cultured in high-sugar DMEM (Jiangsu Kaiji, cat. no. KGL1206-500) and incubated in a humidified incubator at 37 °C, 5% CO_2_. Short-tandem-repeat authentication of the cells used in this study showed that there was no misidentification or contamination with other cells. The cells used in this research were excluded from *Mycoplasma* contamination.

### Plasmids and lentivirus

The ovalbumin (OVA)-overexpressing MC38 lines were generated by lentivirus (Hycyte Biotechnology, cat. no. CONLVOE209). The lentiviruses for small hairpin (sh)RNA targeting mouse STING or negative control (NC), and small interfering (si)RNA targeting HMGCR were purchased from General Bio. The cells were seeded in six-well plates, and transfection experiments were conducted once the cells reached 50–60% confluence. Cells were transfected with shRNA or siRNA by Lipofectamine 2000 (Invitrogen, cat. no. 11668027) according to the manufacturer’s protocol. Transfection efficiency was assessed via quantitative polymerase chain reaction (qPCR) and western blotting 48 h after transfection. The sequences were as follows:

Mouse shNC: 5′-TTCTCCGAACGTGTCACGT-3′;

Mouse shSTING: 5′-AGAGGTCACCGCTCCAAATAT-3′;

Mouse siNC: 5′-UUCUCCGAACGUGUCACGUTT-3′;

Mouse siHMGCR#1:5′-CGGCAGGACCCGTCTTTAAAGTGAT-3′;

Mouse siHMGCR#2:5′-GACCCGTCTTTAAAGTGATGGAGAT-3′;

Mouse siHMGCR#3: 5′-GATGGAGATGTAGGCTGCAAATTGA-3′;

Human siNC: 5′-UUCUCCGAACGUGUCACGUTT-3′;

Human siHMGCR#1: 5′-GATGCATAGCCATCCTGTATATTTA-3′;

Human siHMGCR#2: 5′-ACACGATGCATAGCCATCCTGTATA-3′;

Human siHMGCR#3: 5′-CAGCTTGAAATTATGTGCTGCTTTG-3′.

### Extracellular dsDNA detection

The supernatant containing dsDNA was collected and measured by Quant-iT PicoGreen dsDNA Reagent and Kits (Invitrogen, cat. no. P7589), according to the manufacturer’s protocol^27^.

### Cell proliferation assay

A total of 3000 cells were plated into each well of a 96-well plate with different treatments. Subsequently, the cells were incubated for a period of three consecutive days to assess their viability using the cell counting kit-8 assay (CCK-8) (TargetMol, cat. no. C0005). After the addition of CCK-8, the plates were incubated for a further 2 h, after which the absorption values at 450 nm were measured.

### Colony formation assay

A total of 500 cells were plated into each well of a 12-well plate with different treatments. The cells were incubated for a period of 10 days, after which the culture medium was removed. The colonies were then fixed in 10% formalin solution for 1 h and subsequently stained with 0.1% crystal violet solution at room temperature for 30 min.

### Isolation and activation of CD8^+^ T cells from OT-1 mice or PBMCs

Spleen was collected from 10-week-old OT-1 mice and CD8^+^ T cells were isolated using anti-mouse CD8a (Ly-2) MACS microbeads (Miltenyi Biotec, cat. no. 130-117-044), following the manufacturer’s protocol. Human CD8^+^ T Cells Positive Selection Kit (IPHASE Biosciences, cat. no. 071A103.11) was used to isolate CD8^+^ T cells from peripheral blood mononuclear cells (PBMCs), in accordance with the manufacturer’s protocol. To activate the freshly isolated CD8^+^ T cells in vitro, the cells were cultured in RPMI 1640 medium supplemented with the OVA_257–264_ peptide (InvivoGene, cat. no. vac-sin), anti-CD3/CD28 antibodies (Thermo Fisher Scientific, cat. no. 16-0031-85; Thermo Fisher Scientific, cat. no. 16-0281-85; BioLegend, cat. no. 302934; BioLegend, cat. no. 317326) and IL-2 (Pricella, cat. no. PB180634) for 72 h.

### Co-culture experiment

Tumor cells were initially labeled with carboxy fluorescein diacetate-succinimidyl ester (CFDA-SE) (Beyotime, cat. no. C1031) and seeded at a density of 10^5^ cells per well in a 12-well plate with various treatments. After the removal of the treatments, lymphocytes were added at a tenfold dilution for a 36-h co-culture period. After the co-culture period, the cells were washed with phosphate-buffered saline (PBS), stained with Zombie NIR (BioLegend, cat. no. 77184) for 30 min, washed again and resuspended. Subsequently, the proportion of dead cells was determined through flow cytometry.

### LDH release assay

After a 36-h co-culture of tumor cells and immune cells with varying treatments, the lactate dehydrogenase (LDH) levels in the culture supernatants were quantified in accordance with the manufacturer’s instructions.

### Enzyme-linked immunosorbent assay

Cytokine analysis was conducted using the Mouse IFN-γ ELISA Kit (BioLegend, cat. no. 430804), the Human IFN-γ ELISA Kit (BioLegend, cat. no. 430104), the Mouse IFN-β ELISA Kit (Multi Science, cat. no. EK2236), the Human IFN-β ELISA Kit (Multi Science, cat. no. EK1236) and the Mouse CCL5 ELISA Kit (Multi Science, cat. no. EK2129), in accordance with the instructions provided by the manufacturer.

### Animal experiments

All animal studies were conducted in accordance with the guidelines set forth by the Nanjing Medical University Animal Care and Use Committee and with the approval of the aforementioned committee. Seven-week-old female BALB/c and C57BL/6J Wild-type (WT) mice were purchased from Tande Biotechnology for constructing subcutaneous xenograft models. C57BL/6J CD8-knockout (KO) mice were obtained from Gempharmatech. OT-1 mice were purchased from Jiangsu Wukong Biotechnology. All animal studies adhered to ethical regulations for animal testing and research. A total of 1 × 10^6^ CT26 or MC38 cells were injected subcutaneously into each mouse. Tumor sizes were measured using calipers every 2 or 3 days after the tumor dimensions had reached 100 mm^3^. The tumor volume was calculated according to the following formula: tumor volume = 0.5 × length × width^2^.

### Tumor rechallenge experiment

A total of 1 × 10^6^ CT26 cells were subcutaneously injected into each BALB/c mouse. Once the tumor volume reached 80 mm^3^, the mice were treated with either a blank control or a combination therapy. Upon observing a distinct tumor growth trend, the subcutaneous tumors were surgically removed. After a 1-week recovery period, the mice were rechallenged with CT26 cells on the contralateral side, and tumor growth was monitored. The experimental design is shown in Supplementary Fig. 7a.

### X-ray irradiation experiments in vivo and in vitro

X-ray irradiation exposure was done by using a 6 MV linear accelerator (Elekta Precise) at the Irradiation Center of Jinling Hospital as reported^28,29^.

### Histological analysis

The tissue slices were initially subjected to a 2-h incubation period at 70 °C within an oven. Subsequently, the tissue samples were deparaffinized using a sequential treatment comprising xylene, 100% ethanol, 75% ethanol, 50% ethanol and distilled water. Subsequently, antigen retrieval was conducted using sodium citrate antigen retrieval solution, followed by the inactivation of endogenous peroxidase using 3% hydrogen peroxide. Subsequently, the tissue slices were incubated with 5% goat serum at room temperature for 1 h. Subsequently, the primary antibody was added and the slices were incubated overnight at 4 °C. On the second day, after the removal of the primary antibody, the secondary antibody from the immunohistochemical detection kit (Proteintech, cat. no. KHC0346) was added and the slices were incubated at 37 °C for 1 h. For immunofluorescence (IF), 4′,6-diamidino-2-phenylindole (DAPI) was added to stain the cell nuclei, and the solution was incubated at room temperature for 10 min. Subsequently, the sections were washed and stained for 5 min using a tyramide signal amplification kit (Hunan Aifang Biotechnology, cat. no. AFIHC024). After the diaminobenzidine (DAB) staining, the slides were washed and subsequently treated with hematoxylin, differentiation solution and bluing reagent in a sequential manner. Subsequently, the slides were subjected to a series of dehydration steps involving 50%, 75% and 100% ethanol, followed by xylene. Terminal deoxynucleotidyl transferase-mediated dUTP nick-end labeling (TUNEL) staining was conducted using the TUNEL Bright Red Apoptosis Detection Kit (Vazyme, cat. no. A113-01). Upon completion of the aforementioned steps, the slides were sealed and prepared for microscopic imaging.

### Patient tumor tissues

Tumor tissues were obtained from 30 patients with rectal cancer who were undergoing neoadjuvant radiotherapy at the Eastern Theater General Hospital of the Chinese People’s Liberation Army. Among the patients, 15 demonstrated sensitivities to radiotherapy, while 15 exhibited resistances. Furthermore, samples were obtained from 20 patients at the Jiangsu Provincial People’s Hospital. The CRC pathological sections were subsequently subjected to IHC staining. This study was conducted in accordance with the ethical standards of the Ethics Committee of Jiangsu Provincial People’s Hospital, and informed consent was obtained from patients or their relatives for the use of these clinical materials for research purposes.

### Flow cytometric analysis

First, we took appropriately sized mouse tumor tissue samples and minced them. After mincing, we digested the tumor tissue using a mouse tumor dissociation kit (Miltenyi Biotec, cat. no. 130–0960-730) and then filtered it through a mesh screen. Dead cell populations were excluded using Zombie NIR (BioLegend, cat. no. 77184). Subsequently, staining was conducted using antibodies, including CD45-BV510 (BioLegend, cat. no. 103138), CD3-APC (BioLegend, cat. no. 100236), CD4-BV421 (BioLegend, cat. no. 100438), CD8-FITC (BioLegend, cat. no. 100706) and IFN-γRα-PE (BioLegend, cat. no. 308606), in a sequential manner. Each staining procedure should be conducted at room temperature in the dark for a period of approximately 30 min. To improve the proportion of cells expressing IFN-γ, it is recommended to prestimulate the digested cells with Cell Stimulation Cocktail (Invitrogen, cat. no. 2825902) at 37 °C for 4 h before conducting IFN-γ staining. Ultimately, the cells should be resuspended in PBS and analyzed using a flow cytometer.

### RNA isolation and quantitative real-time PCR

Appropriately sized tumor tissue samples were collected and homogenized thoroughly in TRIzon Reagent (CwBio, cat. no. CW0580S). The homogenized samples was transferred to Eppendorf tubes, chloroform was added and the samples were centrifuged at 12,000 rpm for 10 min. The supernatant was transferred to a new tube and isopropanol was added, followed by incubation for 10 min before centrifugation at 12,000 rpm for another 10 min. The pellet was washed with ethanol and air-dried, then dissolved in DEPC-treated water. Subsequently, the concentration was quantified for reverse transcription using HiScript II Q RT SuperMix for qPCR (Vazyme Biotech, cat. no. R223-01). The next step entails conducting qPCR on the reverse-transcribed cDNA in accordance with the instructions outlined by the manufacturer of ChamQ SYBR qPCR Master Mix (Without ROX) (Vazyme Biotech, cat. no. Q321-02). After the program is completed, calculations are performed on the basis of the CT values.

Primer sequences of mouse were as follows:

HMGCR: forward 5′-AGCTTGCCCGAATTGTATGTG-3′ and reverse 5′-TCTGTTGTGAACCATGTGACTTC-3′;

SQLE: forward 5′-ATAAGAAATGCGGGGATGTCAC-3′ and reverse 5′-ATATCCGAGAAGGCAGCGAAC-3′;

SREBP2 (SREBF2): forward 5′-GCAGCAACGGGACCATTCT-3′ and reverse 5′-CCCCATGACTAAGTCCTTCAACT-3′;

AMPK: forward 5′-GTCAAAGCCGACCCAATGATA-3′ and reverse 5′-CGTACACGCAAATAATAGGGGTT-3′;

CCL5: forward 5′-TTTGCCTACCTCTCCCTCGA-3′ and reverse 5′-CGACTGCAAGATTGGAGCACT-3′;

CXCL10: forward 5′-CCAAGTGCTGCCGTCATTTTCH -3′ and reverse 5′-GGCTCGCAGGGATGATTTCAAC -3′;

IFNβ: forward 5′-CAGCTCCAAGAAAGGACGAACC-3′ and reverse 5′-GGCAGTGTAACTCTTCTGCAT-3′;

IFNγ: forward 5′-GCCACGCACAGTCATTGA-3′ and reverse 5′-TGCTGATGGCCTGATTGTCTT-3′;

CXCL9: forward 5′-GGAGTTCGAGGAACCCTAGTG-3′ and reverse 5′-GGGATTTGTAGTGGATCGTGC-3′;

GZMB: forward 5′-TCTCGACCCTACATGGCCTTA-3′ and reverse 5′-TCCTGTTCTTTGATGTTGTGGG-3′;

PRF1: forward 5′-CTGCCACTCGGTCAGAATG-3′ and reverse 5′-CGGAGGGTAGTCACATCCAT-3′;

Actin: forward 5′-CGCGAGAGAAGATGACCCAGATC-3′ and reverse 5′-GCCAGAGGCGTACAGGGATA-3′.

Primer sequences of human were as follows:

HMGCR: forward 5′-TGATTGACCTTTCCAGAGCAAG-3′ and reverse 5′-CTAAAATTGCCATTCCACGAGC-3′;

SQLE: forward 5′-TGACAATTCTCATCTGAGGTCCA-3′ and reverse 5′-CAGGGATACCCTTTAGCAGTTTT-3′;

SREBP2 (SREBF2): forward 5′-AACGGTCATTCACCCAGGTC-3′ and reverse 5′-GGCTGAAGAATAGGAGTTGCC-3′;

AMPK: forward 5′-TTGAAACCTGAAAATGTCCTGCT-3′ and reverse 5′-GGTGAGCCACAACTTGTTCTT-3′;

CCL5: forward 5′-CTGCTTTGCCTACATTGCCC-3′ and reverse 5′-TCGGGTGACAAAGACGACTGC-3′;

CXCL10: forward 5′-AGCAGAGGAACCTCCAGTCTH-3′ and reverse 5′-AGGTACTCCTTGAATGCCACT-3′;

IFNβ: forward 5′-ACGCCGCATTGACCATCTAT-3′ and reverse 5′-GTCTCATTCCAGCCAGTGCT-3′;

Actin: forward 5′-CGCGAGAGAAGATGACCCAGATC-3′ and reverse 5′-GCCAGAGGCGTACAGGGATA-3′.

### Western blot

Cells were collected in 1.5-ml Eppendorf tubes and washed twice with precooled PBS, and the cells were lysed with RIPA (Beyotime, cat. no. P0013B) lysis and protease inhibitor (Beyotime, cat. no. P1006) on ice for 30 min. Afterward, the lysate was centrifuged at 12,000 rpm for 15 min at 4 °C, and the supernatant was collected. Protein quantification of the cell lysate was performed using the BCA Protein Quantification Kit. After quantification, protein sampling buffer was added and mixed well. The mixture was then boiled in a water bath for 5 min and subjected to SDS–PAGE electrophoresis, and the resulting protein was transferred to a polyvinylidene fluoride membrane. After the transfer, the polyvinylidene fluoride membrane was covered with 5% skim milk powder and incubated at room temperature for 2 h. Then, the specific primary antibody was added and incubated overnight at 4 °C. The following day, the secondary antibody (either rabbit or mouse species) was added and incubated at room temperature with shaking for 2 h. Finally, the results were assessed using an ECL kit (Tanon, cat. no. 180-501) according to the manufacturer’s instructions.

### Untargeted metabolomics analysis

CT26 tumors were randomly collected 13 days after IR for subsequent untargeted metabolomics analysis. Deep sequencing was performed by Metware Metabolism Bio-technology. Deep analysis was performed on Metware Cloud (https://cloud.metware.cn).

### RNA-seq assay

CT26 tumors were randomly collected 11 days after IR for subsequent RNA sequencing (RNA-seq) analysis. Total RNA was extracted using TRIzol for cDNA library construction. Subsequently, deep sequencing was performed by Novogene via the Illumina HiSeq platform. Differential gene expression^30^ analysis was carried out utilizing the edgeR package within the R software environment, with visualization achieved through the heatmap package. Differentially expressed genes were defined as genes exhibiting a fold change exceeding 2, coupled with an adjusted *P* value below 0.05. The signatures of T cells and natural killer (NK) cells are determined on the basis of the gene composition from public databases, and their signature scores are defined by scaling all expressions.

### HLA typing assay

HCT116 cells and peripheral blood of volunteers were tested for HLA typing by iGeneTech.

### Statistical analysis

Statistical analysis was performed using GraphPad Prism 9.5. All results were shown as the mean ± s.e.m. Student’s *t*-test was utilized to assess significant differences between the two groups, and analysis of variance was used to analyze different variables in multiple groups. Statistical significance was set at *P < 0.05; **P < 0.01; ns, not significant.

## Results

### Multi-omics analysis suggests that radiotherapy promotes cholesterol biosynthesis in tumors

**Fig. 1:**
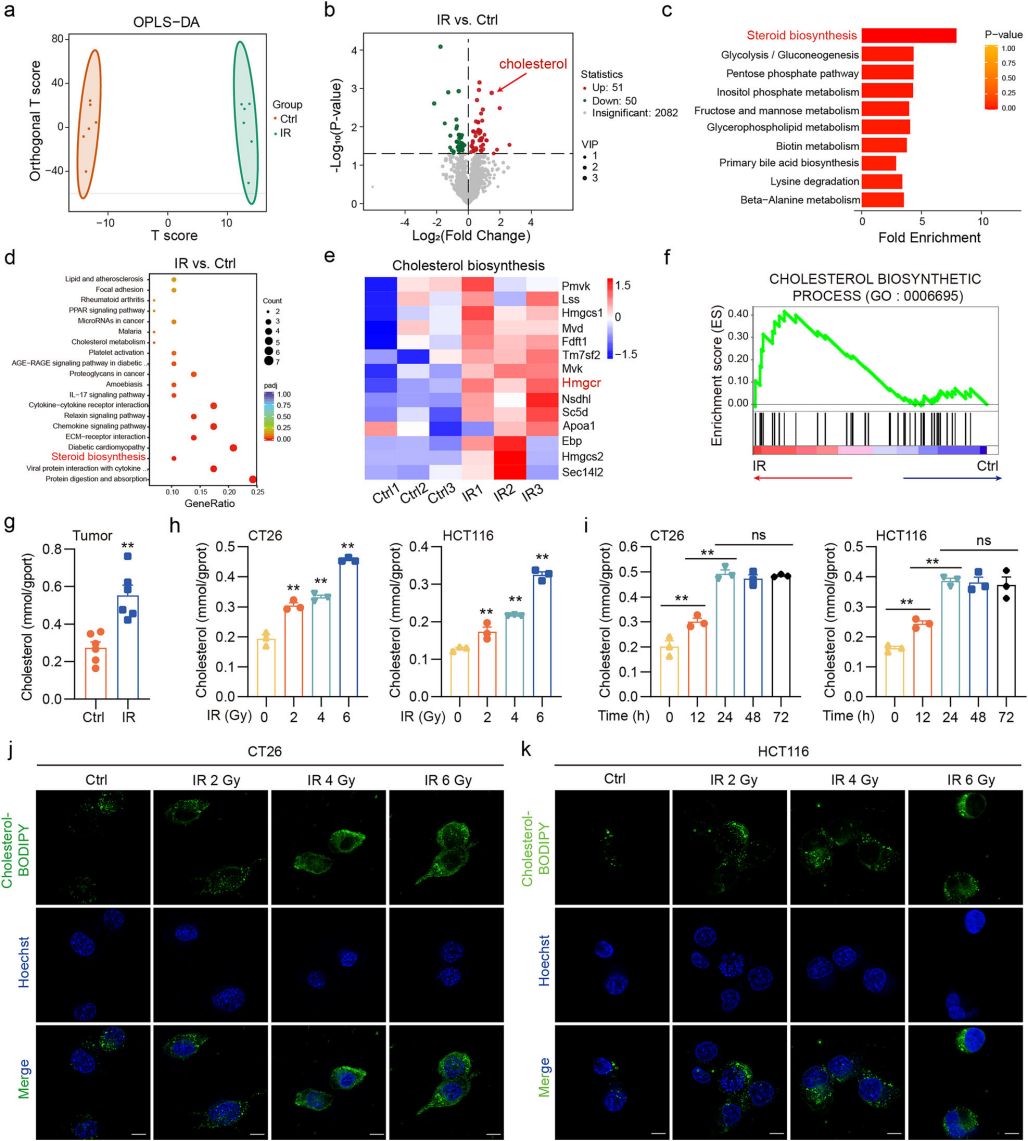
Multi-omics analysis suggests radiotherapy promotes cholesterol synthesis in tumor tissue. **a** Plot of the orthogonal projections to latent structures discriminant analysis (OPLS-DA) score for metabolomics in the two groups. **b** Volcano plots of the metabolomics with significant changes after radiotherapy. **c** Bar chart of metabolite sets enrichment analysis (MSEA) of differential metabolites in the two groups. **d** The top 20 significantly enriched pathways in GO terms for biological process (BP). **e** Heatmap of log_2_ fold change (FC) to depict the gene expression associated with cholesterol biosynthesis. **f** GSEA of cholesterol biosynthetic process (GO: 0006695) between the two groups. **g** The cholesterol levels in CT26 tumor tissues on the 10th day after local 6 Gy irradiation. **h** The cholesterol levels in CT26 and HCT116 cells at 24 h after exposure to 0, 2, 4 and 6 Gy irradiation. **i** The cholesterol levels in CT26 and HCT116 cells at different times (0, 12, 24, 48 and 72 h) after exposure to 6 Gy irradiation. **j**, **k** At 24 h after exposure to 0, 2, 4 and 6 Gy, CT26 (**j**) and HCT116 cells (**k**) were labeled with Hoechst (blue) and cholesterol-BODIPY (green). Scale bar, 10 μm. **P < 0.01; *P < 0.05; ns, not significant.

Recent studies have indicated that radiotherapy promotes metabolic reprogramming in tumors^31–33^. To investigate the effects of radiotherapy on the metabolic and transcriptional changes in tumor tissues, we performed RNA-seq and untargeted metabolomics analyses on CT26 tumors (Supplementary Fig. 1a–c). Radiotherapy led to substantial alterations in metabolic processes and metabolite profiles, with metabolomics analysis revealing a significant increase in cholesterol levels after treatment (Fig. 1a, b). Integrated metabolomic and transcriptomic analyses indicated significant enrichment of the steroid biosynthesis pathway (Fig. 1c, d). Notably, clustered heatmap analysis of genes involved in cholesterol synthesis demonstrated that radiotherapy markedly promoted cholesterol biosynthesis (Fig. 1e). Moreover, the results of the gene set enrichment analysis (GSEA) indicated that the cholesterol biosynthesis and metabolism pathways were significantly enriched in the radiotherapy group (Fig. 1f and Supplementary Fig. 1d, e). Here, we observed an increase in cholesterol levels in CT26 tumor tissues after radiotherapy (Fig. 1g); the phenomenon was also confirmed in CRC cells (Fig. 1h, i). Using confocal microscopy, we further demonstrated increased cholesterol levels in tumor tissues and CRC cells after radiation exposure (Fig. 1j,k and Supplementary Fig. 1f, g). Taken together, our study reveals that radiotherapy significantly alters the metabolic and transcriptional profiles in tumor tissues by promoting the biosynthesis of cholesterol.

### Radiation exposure promotes the upregulation of HMGCR expression and increased cholesterol synthesis in tumors

**Fig. 2:**
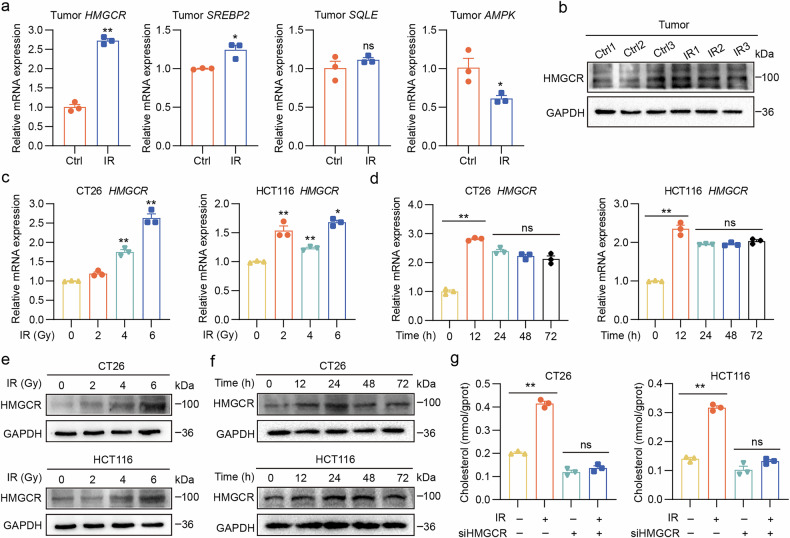
Radiation exposure precipitates cholesterol synthesis by upregulating HMGCR expression in CT26 tumors and CRC cells. **a** The relative expression of HMGCR, SREBP2, SQLE and AMPK mRNA extracted from CT26 tumors was detected by qPCR. **b** The expression of HMGCR protein extracted from CT26 tumors was detected by western blot. **c** The relative expression of HMGCR mRNA extracted from CT26 and HCT116 cells at 24 h after exposure to different doses of irradiation (0, 2, 4 and 6 Gy) was detected by qPCR. **d** The relative expression of HMGCR mRNA extracted from CT26 and HCT116 cells at different times (0, 12, 24, 48 and 72 h) after exposure to 6 Gy irradiation was detected by qPCR. **e** The expression of HMGCR protein extracted from CT26 and HCT116 cells at 24 h after exposure to different doses of irradiation (0, 2, 4 and 6 Gy) was detected by western blot. **f** The expression of HMGCR protein extracted from CT26 and HCT116 cells at different times (0, 12, 24, 48 and 72 h) after exposure to 6 Gy irradiation was detected by western blot. **g** The cholesterol levels in CT26 and HCT116 cells with siHMGCR interference at 24 h after exposure to 6 Gy irradiation. **P < 0.01; *P < 0.05; ns, not significant.

To investigate the underlying cause of this observed increase in cholesterol synthesis, we evaluated the expression levels of several key regulatory genes within the cholesterol synthesis pathway. HMGCR, a crucial enzyme in cholesterol synthesis, was significantly upregulated. However, no discernible alterations were observed in the expression of sterol response element-binding protein 2 (SREBP2) and squalene epoxidase (SQLE). We also noted a slight decrease in the expression of adenosine monophosphate-activated protein kinase (AMPK), a negative regulator of the SREBP family responsible for inhibiting cholesterol synthesis (Fig. 2a). The upregulation of HMGCR levels was confirmed by western blot analyses and IF (Fig. 2b and Supplementary Fig. 1h, i), indicating that HMGCR was elevated at both the protein and transcriptional levels. This result was confirmed in CRC cells (Fig. 2c–f and Supplementary Fig. 1j, k). To ascertain whether HMGCR mediates radiotherapy-induced cholesterol synthesis in CRC cells, we conducted a transfection experiment using siRNAs targeting HMGCR (siHMGCR). The results demonstrated that siRNA transfection effectively reduced HMGCR expression (Supplementary Fig. 1l–o). As anticipated, the levels of cholesterol were found to be unaltered after irradiation in the absence of HMGCR (Fig. 1g). These results indicate a strong correlation between the significant upregulation of HMGCR expression in tumor tissues and CRC cells after radiation exposure and the subsequent promotion of cholesterol biosynthesis.

### Elevated levels of HMGCR and cholesterol diminish the therapeutic efficacy of radiotherapy in patients with CRC

**Fig. 3:**
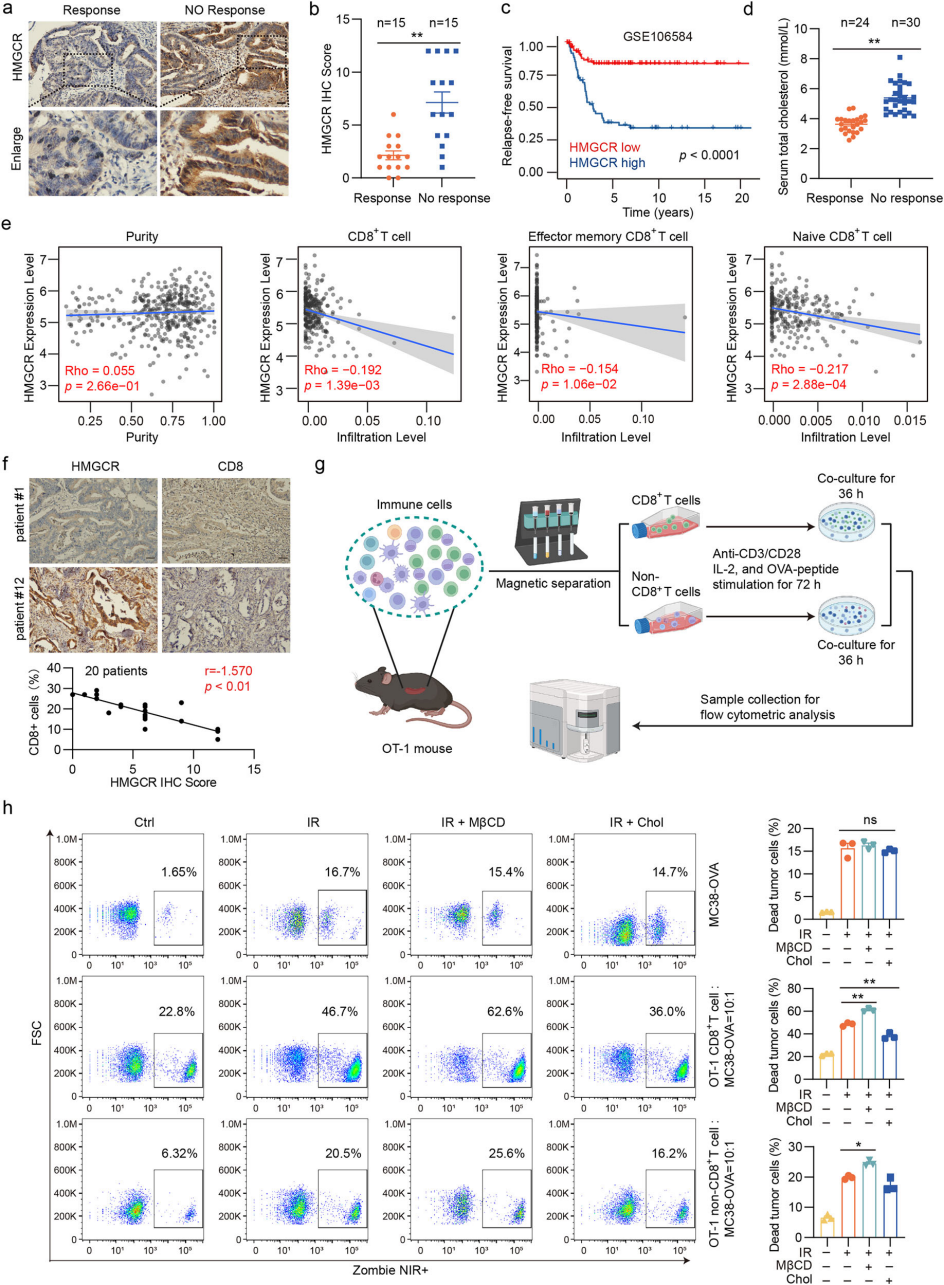
High levels of HMGCR and cholesterol are associated with poor prognosis and reduced response to radiation therapy in patients with CRC. **a**, **b** Representative IHC staining images (**a**) and scatter plot (**b**) comparing HMGCR protein levels (IHC score) between the CRC response group (*n* = 15) and no response group (*n* = 15). **c** Analysis of the cancer genome atlas (TCGA) data (GSE106584) showing that high HMGCR expression correlates with poor survival in a CRC population according to the BEST online database (https://rookieutopia.com/app_direct/BEST/). **d** Clinical cohort data indicating that the serum total cholesterol levels were higher in the radiotherapy nonresponsive group (*n* = 30) compared with the responsive group (*n* = 24). **e** HMGCR expression levels showing a negative correlation with CD8^+^ T cell, effector memory CD8^+^ T cell and naive CD8^+^ T cell according to the TIMER2 online database (http://timer.cistrome.org/). **f** Representative IHC staining images of HMGCR and CD8^+^ T cells in CRC specimens, along with a correlation analysis (*n* = 20). **g** A schematic illustration showing the co-culture of MC38-OVA tumor cells and splenic immune cells from OT-1 mice. **h** MC38-OVA cells with different treatments (control (ctrl), IR 6 Gy, IR + cholesterol 50 μM and IR + MβCD 2 mM) were co-cultured with CD8^+^ T cells or non-CD8^+^ T cells extracted from the spleen of OT-1 mice for a 36-h period, where cholesterol was pretreated for 12 h and MβCD was pretreated for 4 h. The ratio of immune cells to tumor cells was 0:1 and 10:1, respectively, followed by flow cytometry analysis and quantitative analysis. Scale bar, 50 μm. **P < 0.01; *P < 0.05; ns, not significant.

To explore the potential correlation between HMGCR or cholesterol levels and response to radiotherapy in clinical patients, tumor pathology sections were initially collected from 15 patients exhibiting radiotherapy sensitivity and tolerance. HMGCR expression levels were found to be significantly elevated in patients who did not respond to radiotherapy by immunohistochemistry (IHC) (Fig. 3a, b). Furthermore, analysis of the CRC cohort GSE106584 revealed that high HMGCR expression levels correlated with an unfavorable prognosis in patients with CRC (Fig. 3c). Subsequently, we conducted a correlation analysis between total serum cholesterol levels and radiotherapy effectiveness. The results showed that the cholesterol levels were significantly lower in the radiotherapy-sensitive group than in the radiotherapy-tolerant group (Fig. 3d). These findings suggest that high HMGCR and cholesterol levels impair the efficacy of radiotherapy, thereby affecting patient prognosis.

Further bioinformatic analysis revealed a negative correlation between HMGCR expression and the presence of CD8^+^ T cells, effector memory CD8^+^ T cells and naive CD8^+^ T cells, suggesting that HMGCR impedes the infiltration and cytotoxic function of CD8^+^ T cells (Fig. 3e). IHC conducted on pathological sections from 20 patients with CRC confirmed that higher HMGCR levels in tumor tissues were associated with reduced CD8^+^ T cell infiltration (Fig. 3f). Furthermore, we applied CD8a magnetic beads to isolate CD8^+^ T cells from the spleens of OT-1 mice. CD8^+^ T cells from OT-I mice specifically recognize the OVA peptide of amino acids 257–264 (SIINFEKL)^34^.To assess their tumor-killing capacity, CD8^+^ T cells and non-CD8^+^ T cells from the spleens of OT-1 mice were then co-cultured with MC38 cells overexpressing OVA_257–264_ (Fig. 3g and Supplementary Fig. 1p). The results showed that cholesterol inhibited radiotherapy-mediated killing of tumor cells by immune cells, particularly CD8^+^ T cells. This effect was reversed by the depletion of intracellular cholesterol with MβCD (Fig. 3h). Effective tumor killing by CD8^+^ T cells relies on the recognition of tumor-specific antigenic peptides presented by human leukocyte antigen class I (HLA-I) molecules. There are three primary HLA-I loci (HLA-A, HLA-B and HLA-C), each with hundreds of isotypes in humans^35,36^. To ensure histocompatibility, we performed HLA typing on HCT116 cells and PBMCs from volunteers. HCT116 cells were confirmed to express HLA-A0201 (Supplementary Table 1), consistent with previous reports^37^. We then co-cultured HLA-A0201-positive PBMCs (Supplementary Table 2) with HCT116 cells and observed results similar to those obtained in the co-culture of MC38-OVA cells and OT-1 spleen cells (Supplementary Fig. 2a). Thus, our results suggest that elevated HMGCR and cholesterol levels may compromise the response to radiotherapy by suppressing the infiltration and cytotoxic capacity of immune cells.

### Cholesterol impairs radiotherapy-induced cGAS–STING activation and lovastatin rescues this activation in vitro

**Fig. 4:**
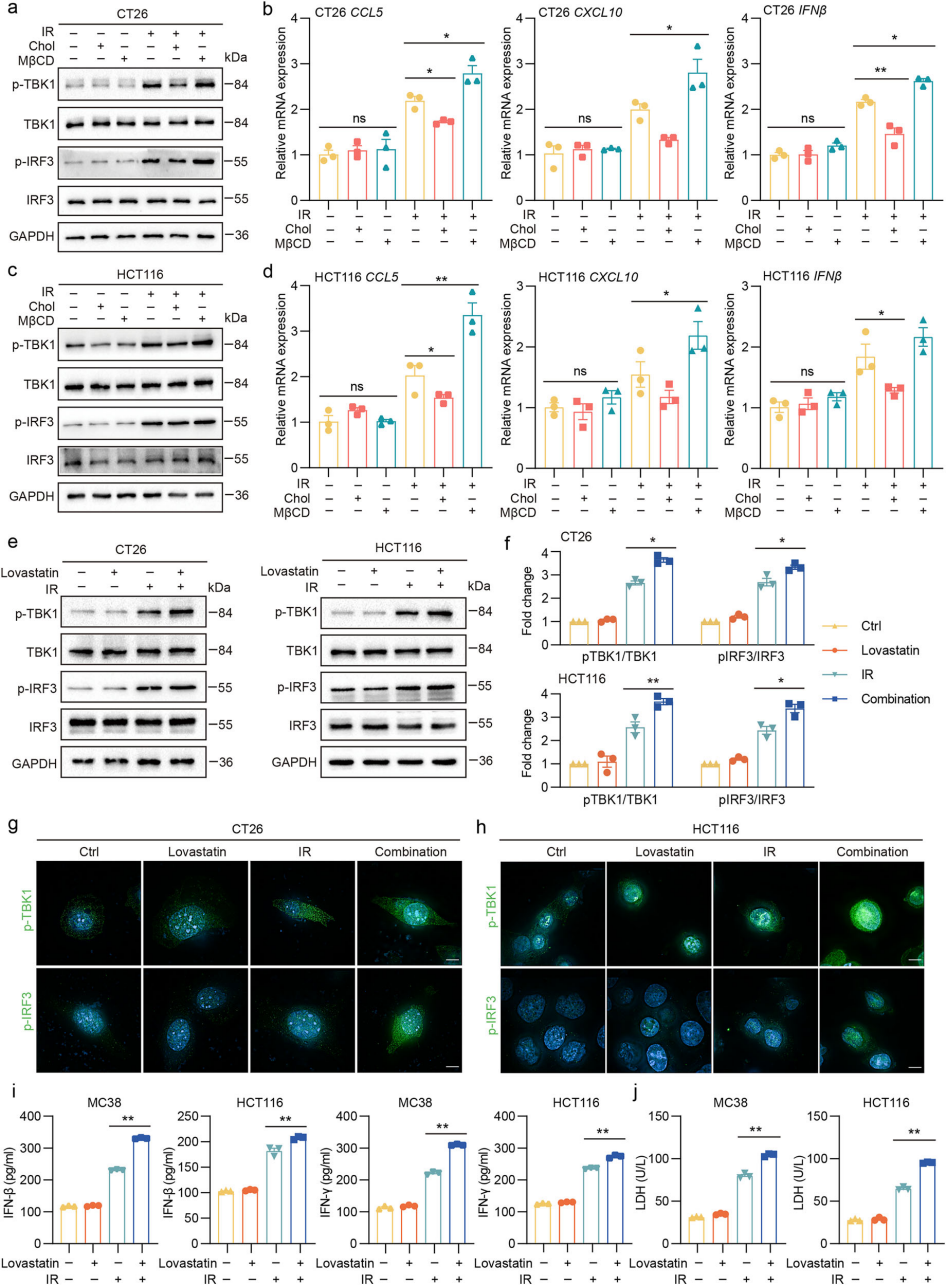
Cholesterol impairs radiotherapy-induced cGAS–STING activation and lovastatin rescues this activation in vitro. **a** The expression of p-TBK1, p-IRF3, TBK1 and IRF3 protein extracted from CT26 cells with different treatments (ctrl, cholesterol 50 μM, MβCD 2 mM, IR 6 Gy, IR + cholesterol and IR + MβCD) and detected by western blot. **b** The relative expression of CCL5, CXCL10 and IFNβ mRNA extracted from CT26 cells was detected by qPCR. **c** The expression of p-TBK1, p-IRF3, TBK1 and IRF3 protein was extracted from HCT116 cells with different treatments (ctrl, cholesterol 50 μM, MβCD 2 mM, IR 6 Gy, IR + cholesterol and IR + MβCD) and detected by western blot. **d** The relative expression of CCL5, CXCL10 and IFNβ mRNA extracted from HCT116 cells was detected by qPCR. **e**, **f** The expression of p-TBK1, p-IRF3, TBK1 and IRF3 protein extracted from CT26 cells and HCT116 cells with different treatments (ctrl, lovastatin 10 μM, IR 6 Gy and IR + lovastatin) was detected by western blot (**e**) and quantitative anslysis (**f**). **g**, **h**, Confocal fluorescence microscopy was conducted on CT26 (**g**) and HCT116 (**h**) cells with different treatments. The cells were labeled with DAPI (blue) and p-TBK1 (green) or p-IRF3 (green). **i** The levels of IFN-β and IFN-γ in the supernatant of co-cultures of MC38-OVA cells and OT-1 mouse spleen cells as well as HCT116 cells and human PBMCs were measured using ELISA. The ratio of immune cells to tumor cells is 10:1. The co-cultures were maintained for 36 h. **j** LDH release assay was performed using the supernatants from co-cultures of MC38-OVA cells and OT-1 mouse spleen cells as well as HCT116 cells and human PBMCs. The ratio of immune cells to tumor cells is 10:1. The co-cultures were maintained for 36 h. Scale bar, 5 μm. **P < 0.01; *P < 0.05; ns, not significant.

These findings prompted us to investigate the underlying mechanisms in detail. Radiotherapy induces dsDNA release, which activates the cGAS–STING pathway and initiates IFN-stimulated gene responses that are crucial for innate immune activation^38^. To examine the influence of cGAS–STING activation of tumor cells on the efficacy of radiotherapy, we established a murine syngeneic tumor model using shNC MC38 or shSTING MC38 cells. Our results demonstrated that shSTING markedly suppressed the impact of radiotherapy on tumor growth (Supplementary Fig. 3a–d). Moreover, shSTING significantly inhibited the IR-mediated activation of the cGAS–STING pathway, suppressed apoptosis and inhibited CD8^+^ T cell infiltration in the tumor microenvironment (Supplementary Fig. 3e–g). The observed reduction in efficacy indicates that the anti-tumor immune response elicited by radiotherapy is dependent primarily on the activation of the cGAS–STING signaling pathway intrinsic to tumor cells. Subsequently, we examined the impact of cholesterol on dsDNA release by measuring dsDNA content in the supernatants of CRC cells under various treatment conditions. Cholesterol did not noticeably impact dsDNA concentration and γH2AX expression, indicating that cholesterol does not affect dsDNA release. Similarly, the level of phosphorylated cGAS (p-cGAS) in CRC cells remained unaffected by cholesterol. However, cholesterol administration resulted in a significant reduction in phosphorylated STING (p-STING) protein levels in CRC cells, suggesting that cholesterol may inhibit STING activation (Supplementary Fig. 4a, b). Cholesterol has been demonstrated to impede cGAS–STING activation by inhibiting the transport of STING from the ER to the Golgi apparatus^26^, and this finding was subsequently confirmed through the execution of confocal experiments (Supplementary Fig. 5). Our findings demonstrated that cholesterol inhibits radiotherapy-induced cGAS–STING activation (Fig. 4a–d).

Given the potential inhibitory effect of cholesterol on cGAS–STING pathway activation, we further explored the impact of reducing cholesterol levels on the efficacy of radiotherapy. We used lovastatin, a statin commonly used to lower cholesterol levels, by targeting HMGCR. The initial experiments involved administering a combination of lovastatin and radiotherapy to the CRC cells. A significant increase in cGAS–STING activation was observed when these two treatments were combined compared with radiotherapy alone. (Fig. 4e, f). Confocal microscopy confirmed this increase in cGAS–STING activation in the CRC cells (Fig. 4g, h). In addition, we investigated the relationship between the combination therapy and immune cell function. ELISA results demonstrated a significant upregulation of IFN-β and IFN-γ levels in the combination treatment group. This elevation in cytokine levels indicated a potentiated immune response within the co-culture system comprising tumor cells and immune effectors (Fig. 4i). These cytokines are essential for an effective anti-tumor immune response in tissues^39^. Interestingly, we observed that the combination treatment did not markedly inhibit tumor cell proliferation compared with radiotherapy alone (Supplementary Fig. 6a, b). However, when co-cultured with immune cells, the combination treatment notably enhanced the cytotoxic effects of immune cells (Fig. 4j and Supplementary Fig. 6c, d). The results demonstrate that the combination of lovastatin and radiotherapy has the potential to enhance anti-tumor immunity in vitro.

### Inhibition of HMGCR by lovastatin sensitizes radiotherapy in the syngeneic model of colon cancer

**Fig. 5:**
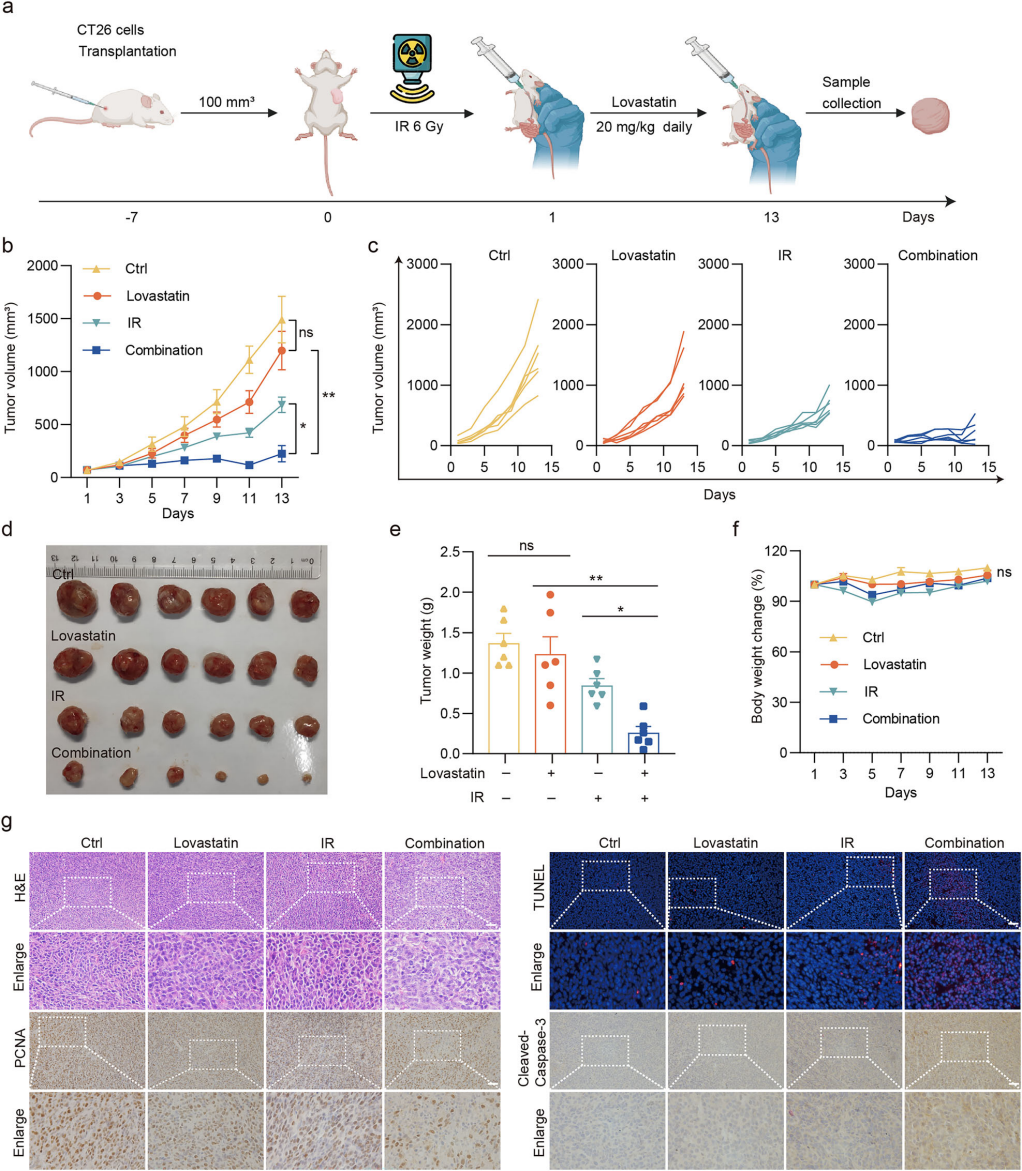
Inhibition of HMGCR by lovastatin sensitizes radiotherapy in mouse subcutaneous transplant tumors. BALB/c mice were subcutaneously inoculated with 1 × 10^6^ CT26 cells. Different treatments commenced when the average tumor volume reached 100 mm^3^, involving a single dose of 6 Gy local tumor irradiation, along with simultaneous oral administration of 20 mg/kg lovastatindaily. **a** A schematic illustration showing the timeline of the drug treatment and assays. **b** Tumor volume (mean ± s.e.m.) was measured after radiotherapy every 2 days. **c** The individual growth profiles of tumors in each group. **d** Solid tumors were separated after the mice were euthanized. **e** Tumor weight (mean ± s.e.m.) was measured after the mice were euthanized. **f** Body weight change (mean ± s.e.m.) was measured after radiotherapy every 2 days. **g** The tumor tissue paraffin sections were subjected to H&E staining, PCNA and Cleaved-Caspase-3 IHC staining, and TUNEL staining. Scale bar, 50 μm. **P < 0.01; *P < 0.05; ns, not significant.

Based on the results above, we evaluated whether lovastatin enhances the effectiveness of radiotherapy in CRC. We established a murine syngeneic tumor model using CT26 cells from BALB/c mice. Our results showed that lovastatin alone did not exert a significant anti-tumor effect. However, when combined with radiotherapy, lovastatin significantly attenuated tumor growth. The average tumor weight in mice treated with combination therapy (0.26 g) was approximately one-fourth of that in mice treated with radiotherapy alone (0.85 g), with no significant reduction in body weight. These findings suggest that lovastatin enhances radiotherapy sensitivity (Fig. 5a–f). Concurrently, the biosafety of the combination therapy was evaluated. The increased tumor tissue necrosis was observed in the combination therapy group, as revealed by hematoxylin and eosin (H&E) staining. IHC staining for proliferating cell nuclear antigen (PCNA) confirmed that combining lovastatin and radiotherapy markedly inhibited tumor cell proliferation in vivo. Moreover, TUNEL and IHC staining for Cleaved-Caspase-3 revealed a substantial increase in apoptosis in the combination therapy group (Fig. 5g).

To explore whether radiotherapy combined with lovastatin produces long-term anti-tumor immune responses in vivo, a tumor rechallenge experiment was performed after subcutaneous tumors had been removed from mice at the end of the initial treatment (Supplementary Fig. 7a). The results demonstrated that the anti-tumor immunity triggered by lovastatin in conjunction with radiotherapy manifested as a long-term response. In the subsequent observation, the tumors in the combination therapy group exhibited slow proliferation, demonstrating a substantial anti-tumor effect (Supplementary Fig. 7b,c). This finding indicates that the combination of lovastatin and radiotherapy not only is effective in the short term but also induces long-term immune memory. Furthermore, the combination therapy did not lead to weight loss in mice in the long term (Supplementary Fig. 7d). H&E staining of major organs (heart, liver, lung and kidney) from mice indicated no significant morphological changes between groups (Supplementary Fig. 7e). A series of hematological tests was also conducted on the blood of mice, and the results demonstrated that the combination therapy did not elicit any notable alterations (Supplementary Fig. 7f). These results fully indicated the sufficient biosafety of the combination therapy. Collectively, these findings underscore the potential of combination therapies as effective strategies for CRC treatment without the added toxicity of lovastatin.

### HMGCR inhibition combined with radiotherapy enhances gene expression and pathway enrichment associated with anti-tumor immunity

**Fig. 6:**
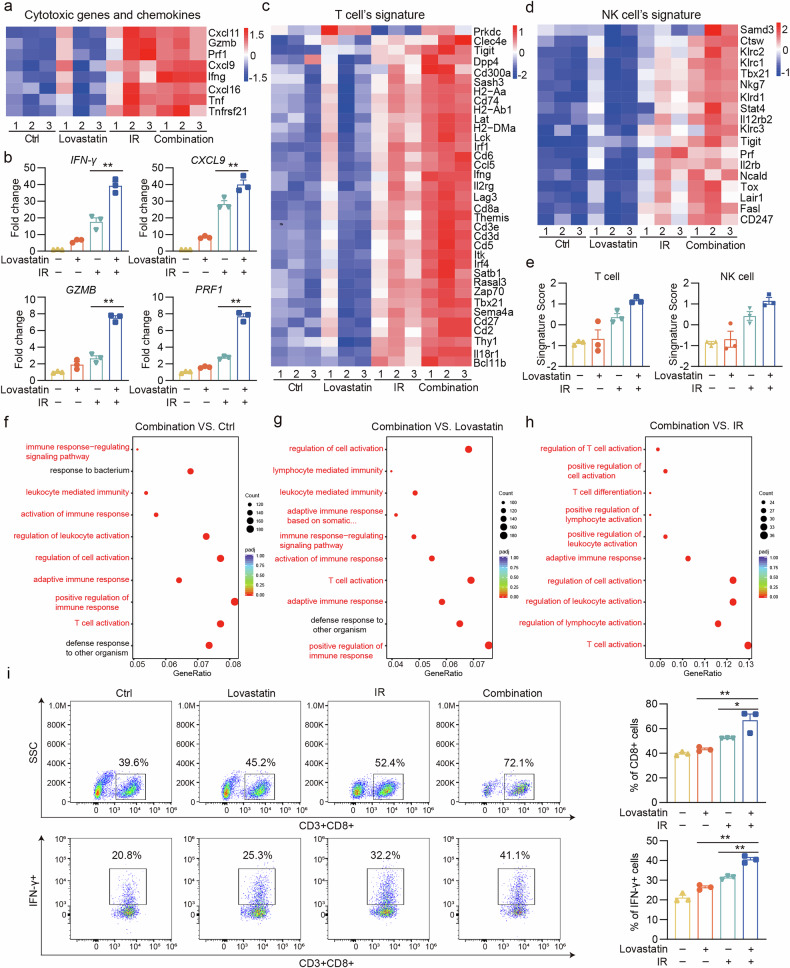
HMGCR inhibition combined with radiotherapy enhances gene expression and pathway enrichment associated with anti-tumor immunity. The transcriptomic analysis was conducted to examine alterations in the tumor microenvironment in the CT26 tumor model. **a**,Heatmap of log_2_FC to depict the gene expression of key cytotoxic genes and chemokines in four groups. **b** Quantitative qPCR validation of selected genes identified by RNA-seq. **c**–**e** Heatmaps of log_2_FC to depict the gene expression of T cell’s signature (**c**) and NK cell’s signature (**d**) in four groups, and signature scores calculated to quantify (**e**). **f**–**h** The top 10 significantly enriched pathways in GO terms for BP in the following comparisons: combination vs. ctrl (**f**), combination vs. lovastatin (**g**), and combination vs. IR (**h**). **i** Examination of CD3^+^ CD8^+^ T cells and IFN-γ secretion in the tumor tissues by flow analysis. **P < 0.01; *P < 0.05.

Although our findings indicated that lovastatin demonstrated a robust radiotherapy sensitization effect in colon cancer, the connection between this phenomenon and the amplified anti-tumor immunity observed in vivo remains uncertain. To address this, we used an RNA-seq assay to analyze the changes in the tumor microenvironment of the CT26 tumor model. Granzyme B (GZMB), IFN-γ and perforin-1 (PRF1), which are associated with inflammation and immunity, are widely recognized for mediating a more potent immune cytotoxic effect^30,40^. The RNA-seq assay revealed a significant upregulation of cytotoxicity-related genes (*GZMB*, *PRF1*, *IFNγ*, tumor necrosis factor and so on) and chemokines (*CXCL9*, *CXCL11* and *CXCL16*) in the combination therapy group (Fig. 6a). This upregulation of several immune-related genes (*GZMB*, *PRF1*, *IFN-γ* and *CXCL9*) was further confirmed by qPCR (Fig. 6b). In addition, we conducted various analyses of immune cell signatures to investigate changes in the tumor microenvironment and found a significant increase in the signature scores of T cells and NK cells in the combination therapy group compared with those in the other three groups (Fig. 6c–e).

Furthermore, Gene Ontology (GO) enrichment analysis of differentially expressed genes between the two groups identified a notable upregulation in several immune-related pathways in the combination therapy group, including T cell activation and differentiation, activation of the immune response, regulation of lymphocyte activation, and lymphocyte-mediated immunity (Fig. 6f–h). These RNA-seq assay results indicated that combination therapy substantially enhances various aspects of tumor immunity, eliciting potent anti-tumor immune responses.

Based on the RNA-seq results, we explored whether diverse treatments affect immune cells within the tumor microenvironment. Flow cytometry assay was performed to explore the quantity and function of tumor-infiltrating lymphocytes in CT26 tumor models. The combination therapy group exhibited an overall increase in the proportion of CD3^+^ T cells. Specifically, a significant increase was observed in the proportion of CD8^+^ T cells compared with that in the other groups, but no significant change was found in the proportion of CD4^+^ T cells. Furthermore, higher levels of IFN-γ were detected in CD8^+^ T cells in the combination therapy group, suggesting that the combination therapy not only promotes the infiltration of CD8^+^ T cells but also significantly enhances their cytotoxic function (Fig. 6i and Supplementary Fig. 8a, b). These findings were validated by IF staining of CD4^+^ T and CD8^+^ T cells (Supplementary Fig. 8c, d) and IHC staining for IFN-γ (Supplementary Fig. 8e). In summary, our findings indicate that the co-administration of lovastatin and radiotherapy significantly boosts the body’s anti-tumor immune response, presenting a promising strategy for combination therapy.

### HMGCR inhibition combined with radiotherapy significantly activates the cGAS–STING pathway in vivo

**Fig. 7:**
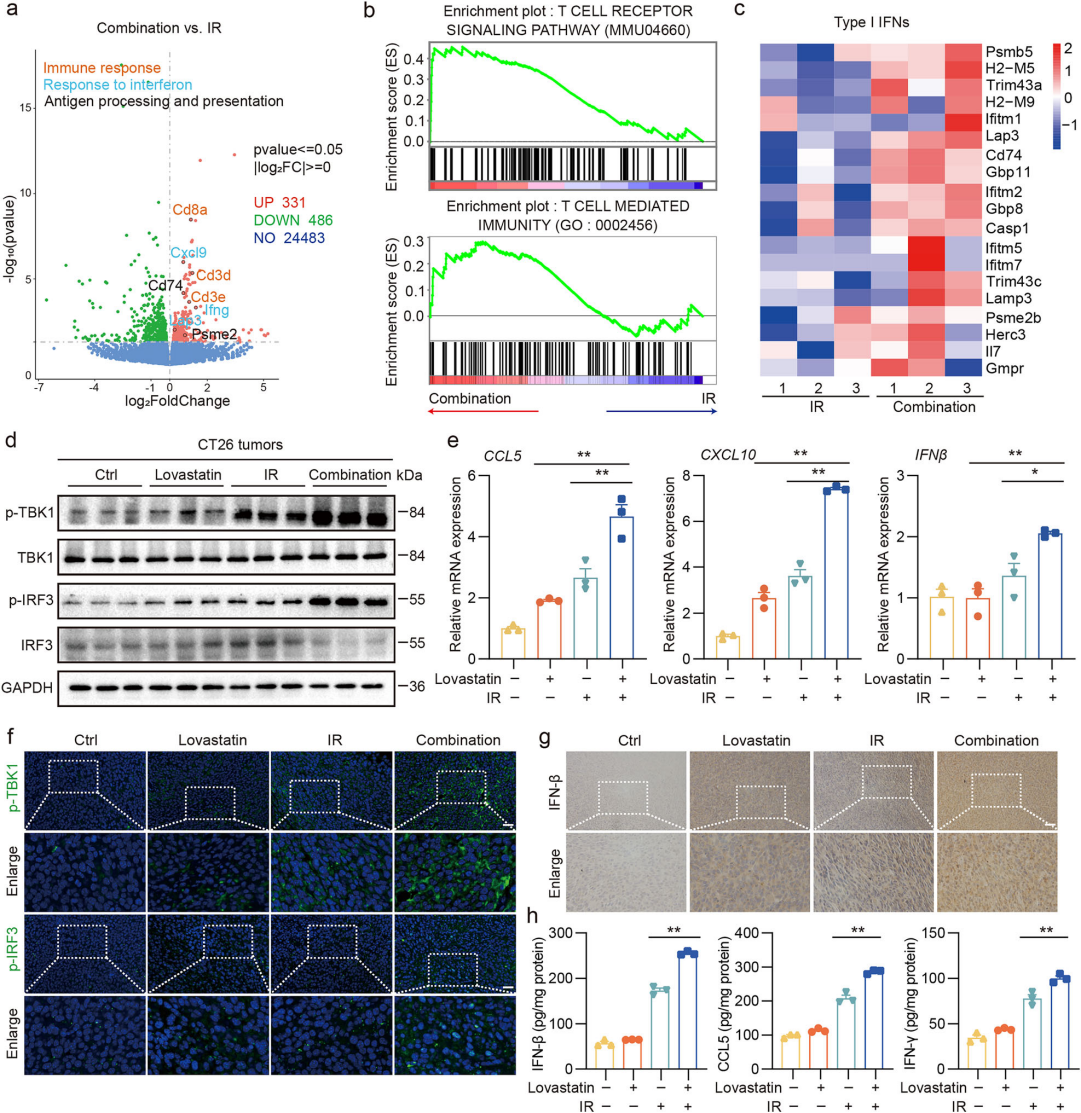
HMGCR inhibition combined with radiotherapy significantly activates the cGAS–STING pathway. **a**, A volcano plot of differentially expressed genes between the combination group and the radiotherapy group. **b** GSEA of the TCR signaling pathway (KEGG: MMU04660) and T-cell-mediated immunity (GO: 0002456) between the combination therapy group and the radiotherapy group. **c** A heatmap of log_2_FC to depict the gene expression associated with type I IFN. **d** The expression of p-TBK1, p-IRF3, TBK1 and IRF3 protein extracted from CT26 tumors was detected by western blot. **e**, The relative expression of CCL5, CXCL10 and IFNβ mRNA extracted from CT26 tumors was detected by qPCR. **f**, Representative IF images of p-TBK1 and p-IRF3. **g**, Representative IHC images of IFN-β. **h**, The levels of IFN-β, CCL5 and IFN-γ protein in tumor tissues were measured via ELISA. Scale bar, 50 μm. **P < 0.01; *P < 0.05.

We constructed an RNA-seq volcano plot to assess differential gene expression between the radiotherapy-only and combination therapy groups. Our analysis identified 817 differentially expressed genes, with 331 upregulated and 486 downregulated genes, in the combination therapy group. Among the 331 upregulated genes, many were linked to positive immune regulation, encompassing functions such as immune response, antigen processing and presentation, and responsiveness to IFNs (Fig. 7a). GSEA revealed increased T cell receptor (TCR) signaling and T-cell-mediated immunity in the combination therapy group compared with the radiotherapy group (Fig. 7b). These results indicate that the addition of lovastatin significantly enhanced the immune modulation of radiotherapy.

To further validate whether lovastatin enhances sensitivity to radiotherapy in a colon cancer xenograft model through the activation of cGAS–STING in vivo, we detected a substantial increase in the expression of genes associated with the cGAS–STING signaling pathway in the combination therapy group compared with the radiotherapy group (Fig. 7c). Immunoblotting revealed that the combination treatment significantly induced the phosphorylation of TANK-binding kinase 1 (TBK1) and interferon regulatory factor 3 (IRF3) without affecting their total protein levels (Fig. 7d). In addition, qPCR showed augmented expression of CCL5, CXCL10 and IFNβ in the combination group (Fig. 7e). IF and IHC experiments demonstrated a significant increase in the expression of phosphorylated TBK1 (p-TBK1), phosphorylated IRF3 (p-IRF3) and IFN-β within the tumor tissue after combination therapy (Fig. 7f, g). ELISA results indicated higher IFN-β, CCL5 and IFN-γ levels, revealing enhanced tumor immunomodulation after the combined treatment (Fig. 7h). These results collectively demonstrate that adding lovastatin significantly enhanced the activation of the cGAS–STING pathway induced by radiotherapy in vivo.

### STING pathway inhibition reverses the HMGCR inhibition-mediated radiotherapy sensitization of colon cancer in vivo

**Fig. 8:**
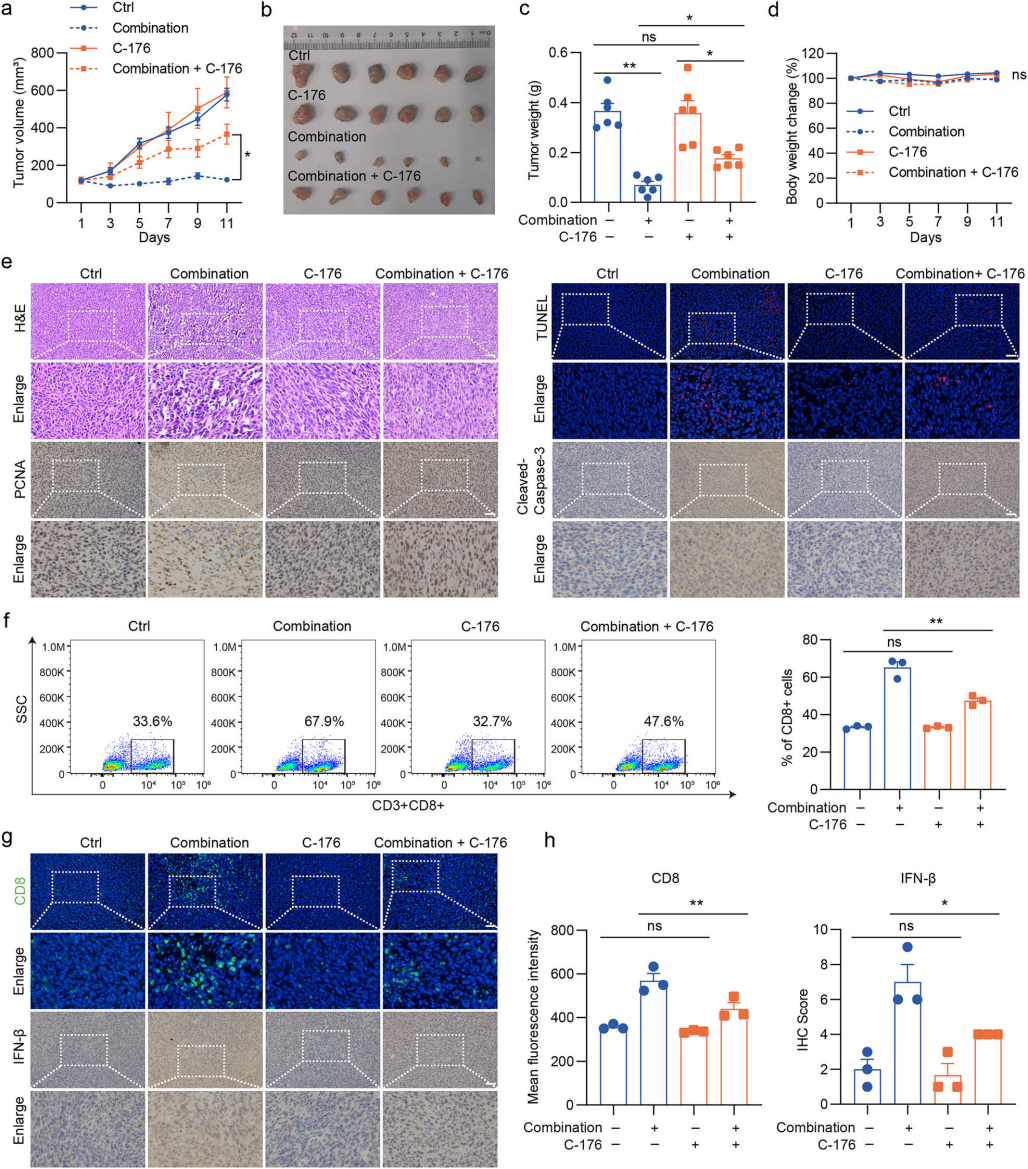
STING pathway inhibition partly reverses the HMGCR inhibition-mediated radiotherapy sensitization of colon cancer in vivo. C57BL/6J mice were subcutaneously inoculated with 1 × 10^6^ MC38 cells. Different treatments commenced when the average tumor volume reached 100 mm³, involving a single dose of 6 Gy local tumor irradiation, along with simultaneous oral administration of 20 mg/kg lovastatin or ctrl daily. C-176 (14.4 mg/kg, daily) was administered via intraperitoneal injection. **a**, Tumor volume (mean ± s.e.m.) was measured after radiotherapy every 2 days. **b**, Solid tumors were separated after the mice were euthanized. **c**, Tumor weight (mean ± s.e.m.) was measured after the mice were euthanized. **d**, Body weight change (mean ± s.e.m.) was measured after radiotherapy every 2 days. **e**, The tumor tissue paraffin sections were subjected to H&E staining, PCNA and Cleaved-Caspase-3 IHC staining, and TUNEL staining. **f**, Examination of CD3^+^ CD8^+^ T cells in the tumor by flow cytometry analysis. **g**, **h**, Representative IF images of tumor-infiltrating CD8^+^ T cells and IHC images of IFN-β (**g**) and quantitative analysis (**h**). Scale bar, 50 μm. **P < 0.01; *P < 0.05; ns: not significant.

To further explore the impact of lovastatin on cGAS–STING pathway-mediated anti-tumor immunity, we used the cGAS–STING pathway inhibitor C-176 in a murine syngeneic tumor model. The transplantation experiment revealed that lovastatin combined with radiotherapy achieved an 80% inhibition rate in C57 mice, consistent with results previously documented in BALB/c mice. Notably, C-176 did not affect tumor growth in mice but partially reversed the effects observed in the combination group, leading to a reduction in the tumor inhibition rate from 80% to 50% (Fig. 8a–d). In addition, we observed that the inclusion of C-176 reversed the tissue necrosis-, antiproliferative- and apoptosis-promoting effects induced by the combination therapy (Fig. 8e). These findings suggest that STING inhibition partly reverses the sensitizing effect of lovastatin in colon cancer radiotherapy. Significantly, C-176 reduced IFN-β expression levels and the infiltration of CD8^+^ T lymphocytes within the tumor microenvironment in the combination therapy group (Fig. 8f–h). These results led us to consider whether the radio-sensitizing effect of lovastatin on colon cancer is linked to CD8^+^ T lymphocytes.

### CD8 KO partially reverses HMGCR inhibition-mediated radiotherapy sensitization of colon cancer in vivo

**Fig. 9:**
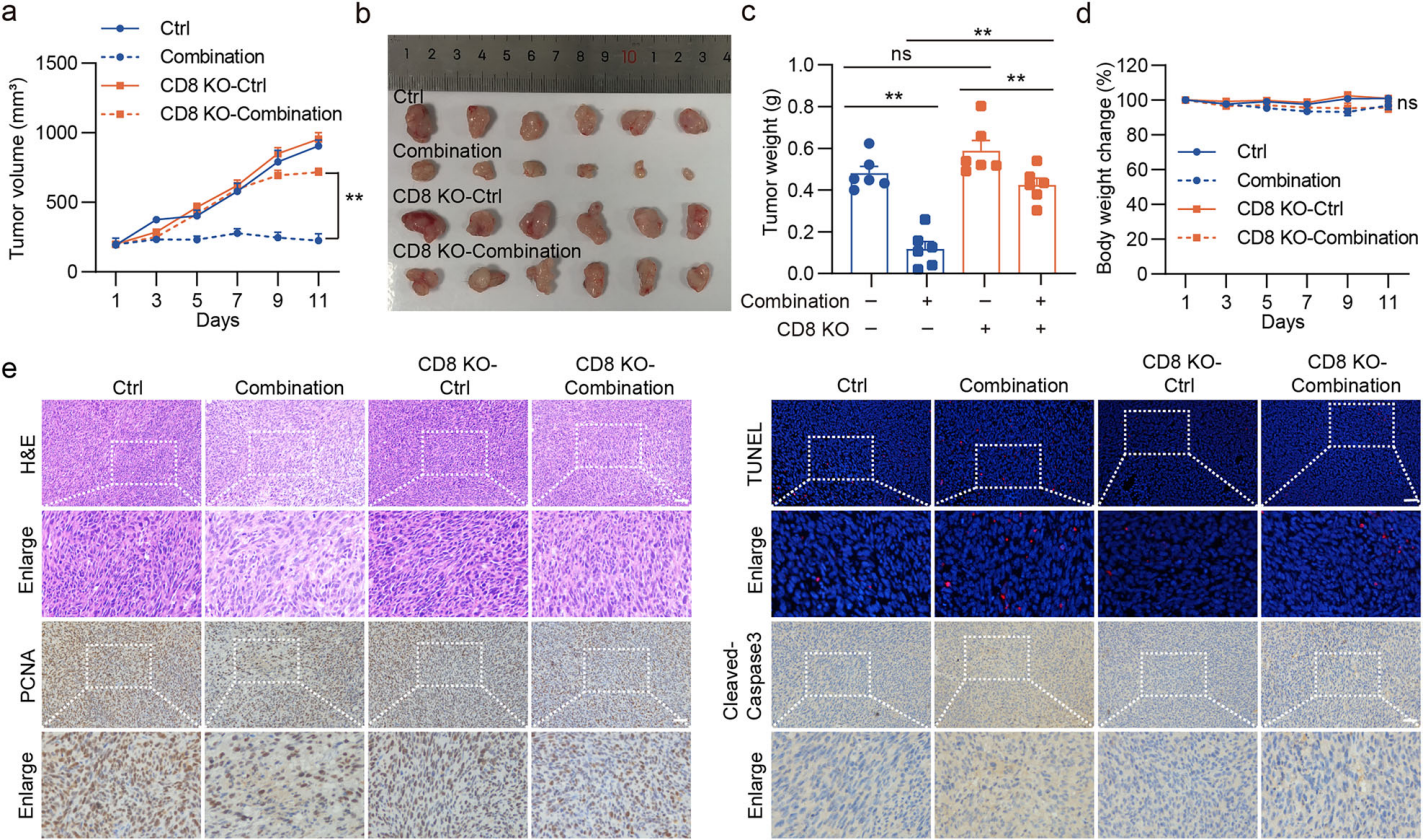
CD8 KO partly reverses the HMGCR inhibition-mediated radiotherapy sensitization of colon cancer in vivo. C57BL/6J WT and CD8-KO mice were subcutaneously inoculated with 1 × 10^6^ MC38 cells. Different treatments commenced when the average tumor volume reached 100 mm³, involving a single dose of 6 Gy local tumor irradiation, along with simultaneous oral administration of 20 mg/kg lovastatin daily. **a**, Tumor volume (mean ± s.e.m.) was measured after radiotherapy every 2 days. **b**, Solid tumors were separated after the mice were euthanized. **c**, Tumor weight (mean ± s.e.m.) was measured after the mice were euthanized. **d**, Body weight change (mean ± s.e.m.) was measured after radiotherapy every 2 days. **e**, The tumor tissue paraffin sections were subjected to H&E staining, PCNA and Cleaved-Caspase-3 IHC staining, and TUNEL staining. Scale bar, 50 μm. **P < 0.01; *P < 0.05; ns, not significant.

We further validated the role of CD8^+^ T lymphocytes in augmenting the efficacy of lovastatin-mediated radiotherapy in colon cancer. We conducted subcutaneous transplantation experiments using WT and CD8-KO mice. A diminished sensitizing effect of lovastatin on radiotherapy was observed in CD8-KO mice, with a tumor inhibition rate of 28.6%, approximately half of that observed in the WT group (77.1%) (Fig. 9a–d). Similarly, mirroring the outcomes observed with C-176 administration, combination therapy effects, such as tissue necrosis attenuation, reduced proliferation and increased apoptosis, were significantly diminished in CD8-KO mice compared with WT mice (Fig. 9e). This experimental evidence led to the conclusion that lovastatin enhances the sensitivity of colon cancer cells to radiotherapy by intensifying the activation of the cGAS–STING pathway, facilitating the type I IFN signaling and boosting the infiltration and cytotoxicity of CD8^+^ T cells.

## Discussion

Radiotherapy is an essential treatment modality for CRC, particularly in cases of rectal and metastatic CRC^41^. Despite its importance, over 70% of patients with CRC exhibit poor responsiveness to radiotherapy, a phenomenon attributed to a variety of factors that ultimately lead to both intrinsic and acquired resistance^42^. Lipid metabolism has been found to contribute to radiotherapy resistance^43–45^. Here, our study revealed a novel connection between radiotherapy and cholesterol biosynthesis in tumors. We found that HMGCR, the first rate-limiting enzyme that regulates cholesterol biosynthesis, increases rapidly in response to radiation, leading to increased cholesterol synthesis. Furthermore, we showed that HMGCR inhibitors may enhance radiotherapy efficacy by boosting anti-tumor activity through further activation of the cGAS–STING pathway, which promotes the infiltration and function of CD8^+^ T cells.

Cholesterol biosynthesis and metabolism are closely correlated with various physiological and pathological states of the body^46–50^. The role of cholesterol in regulating anti-tumor immunity has been the subject of considerable research in recent years, yet findings remain inconsistent. For instance, some studies indicate that tumor cells utilize elevated intracellular cholesterol levels to facilitate T cell depletion and immune evasion, thereby circumventing tumor immune surveillance^51,52^. Conversely, other studies have demonstrated that elevated cholesterol levels in the plasma membrane of CD8^+^ T cells can enhance the signaling capacity of TCR and improve their anti-tumor activity^53,54^. Our study suggests that elevated total serum cholesterol levels may impair the responsiveness of patients with CRC to radiotherapy. Furthermore, increased cholesterol synthesis impedes CD8^+^ T cell infiltration into the tumor microenvironment, which may affect the prognosis of patients with CRC. Notably, the cytotoxic function of CD8^+^ T cells was significantly compromised; however, non-CD8^+^ immune cells were also affected^55^. This may be attributed to STING activation, which not only enhances CD8^+^ T cell function but also promotes dendritic cell activation and antigen presentation^56^. In addition, STING activation induces chemokine production, facilitating NK cell infiltration and activation^57^, reduces regulatory T cell levels in the tumor microenvironment, suppresses the immunosuppressive function of myeloid-derived suppressor cells^58^ and drives tumor-associated macrophages toward an M1-like phenotype^59^, thereby enhancing their anti-tumor activity. In light of these findings, this study concludes that cholesterol plays a role in inhibiting anti-tumor immune responses to radiotherapy.

The cGAS–STING pathway plays a crucial role in initiating the anti-tumor immune response. Radiotherapy has been reported to activate the cGAS–STING pathway, thereby enhancing anti-tumor immunity^60^. Our results indicate that cholesterol inhibits radiotherapy-induced STING activation. Thus, we hypothesized that combining cholesterol-lowering drugs with radiotherapy in treating patients with CRC could potentially enhance cGAS–STING activation, resulting in increased anti-tumor activity. Statins are medications that lower lipid levels via inhibition of cholesterol production by blocking HMGCR^61^. This mechanism makes statins uniquely valuable in cancer therapy, and numerous experimental and clinical studies have confirmed their anti-cancer effects^43,62^. In this study, we found that HMGCR inhibition significantly potentiated the efficacy of radiotherapy, without the added toxicity of HMGCR inhibition. Significant enhancement of cGAS–STING activation was observed after HMGCR inhibition combined with radiotherapy in vivo and in vitro. Furthermore, RNA-seq analysis revealed that this combination therapy significantly upregulated gene expressions and enriched pathways associated with anti-tumor immunity. In addition, flow cytometry and IF showed increased CD8^+^ T cell infiltration and function in the tumor microenvironment of the combination therapy group. Moreover, we suggested a partial reversal of the anti-tumor effects and CD8^+^ T cell infiltration within the combination therapy group upon administration of the STING inhibitor C-176. A partial reversal of the combined effect was observed in the CD8-KO mice. Consequently, this evidence suggests that HMGCR inhibition enhances CD8^+^ T cell infiltration and cytotoxicity through cGAS–STING activation, leading to robust anti-tumor immunity and increased sensitivity of colon cancer cells to radiotherapy.

Our study also has certain limitations. For instance, we have not fully elucidated how cholesterol precisely regulates the cGAS–STING pathway or how lovastatin influences other immune cells in the tumor microenvironment. Future research is needed to explore these complex molecular mechanisms and validate the clinical application potential of these findings in clinical trials.

## Supplementary information

Supplementary Information

Supplementary Tables 1 and 2
