# Supplementary material for: Cholesterol biosynthesis induced by radiotherapy inhibits cGAS–STING activation and contributes to colorectal cancer treatment resistance
Source: Exp Mol Med. 2025 May 12;57(5):1089–105. doi: 10.1038/s12276-025-01457-6 (PMC12130323; doi:10.1038/s12276-025-01457-6)
Supplement: Supplementary file 1 — Supplementary Information [file 12276_2025_1457_MOESM1_ESM.pdf]

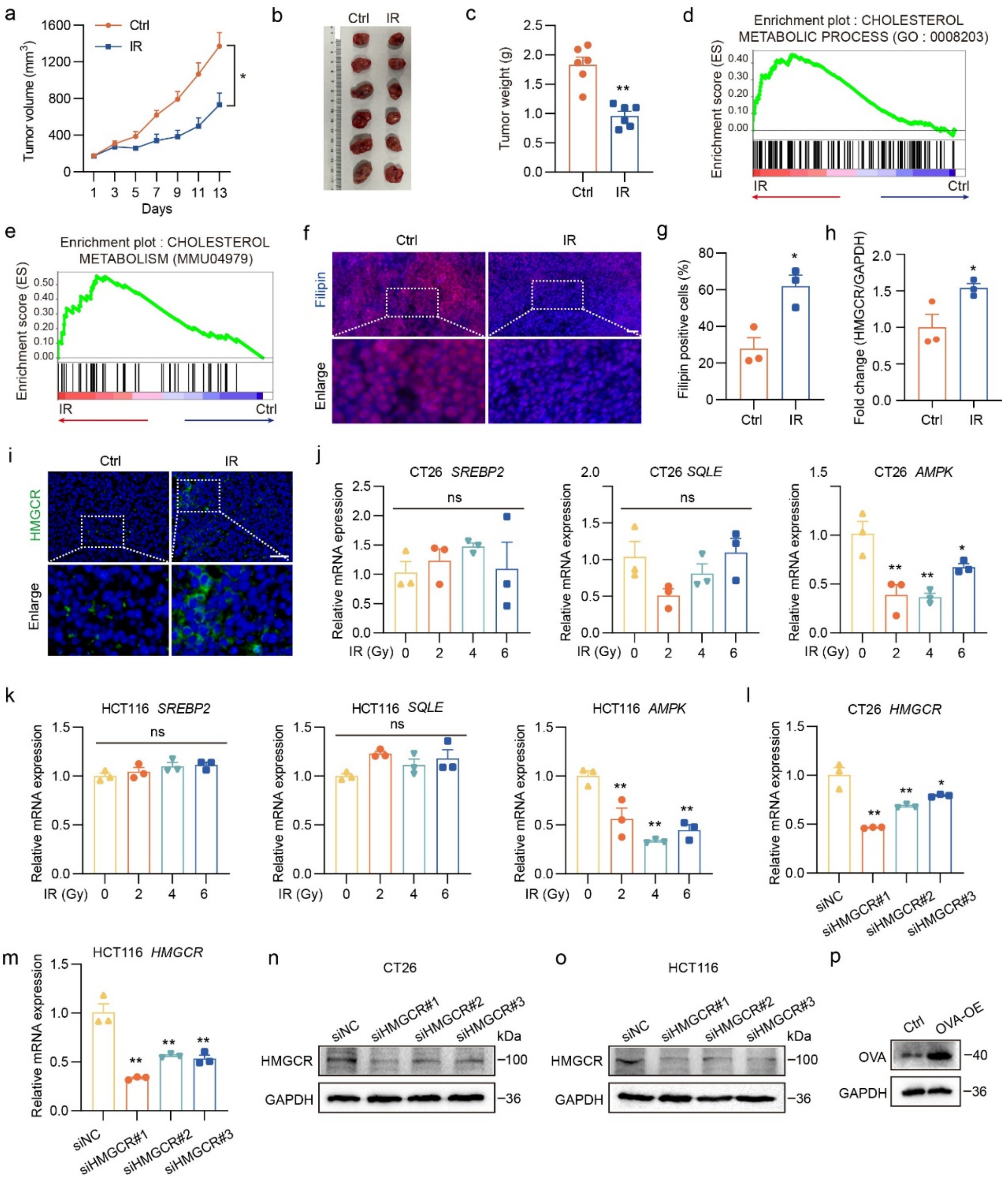

Supplementary Fig. 1 Radiation exposure precipitates cholesterol synthesis through HMGCR expression in CT26 tumor tissues and CRC cells. (a) Tumor volume (mean ± SEM) was measured after radiotherapy every two days. **(b)** Solid tumors were separated after the mice were sacrificed. **(c)** Tumor weight (mean ± SEM) was measured after the mice were sacrificed. **(d)** GSEA of cholesterol metabolic process (GO: 0008203) between the two groups. **(e)** GSEA of cholesterol metabolism (MMU04979) between the two groups. **(f)** Tumor tissue slices from untreated or 6 Gy irradiated mice were stained with filipin III (blue) to visualize cell-associated

cholesterol. **(g)** Quantitative analysis of filipin III staining between the ctrl group and the radiotherapy group. **(h)** Quantitative analysis of HMGCR/GAPDH expression detected by western blot. **(i)** Representative IF images of HMGCR in CT26 tumor tissue from the ctrl group and the radiotherapy group. **(j, k)** Relative expression of *SREBP2*, *SQLE*, and *AMPK* mRNA extracted from CT26 cells (j) and HCT116 cells (k) was detected by qPCR. **(l, m)** The expression of *HMGCR* after the interference of 100 nM siRNA targeting HMGCR was examined by qPCR in CT26 (l) and HCT116 cells (m). **(n, o)** The expression of HMGCR after the interference of 100 nM siRNA targeting HMGCR was examined by western blot in CT26 (n) and HCT116 cells (o). **(p)** The expression of OVA was examined by western blot in MC38 cells. Scale bar, 50 μm. $**P < 0.01$; $*P < 0.05$; ns: not significant.

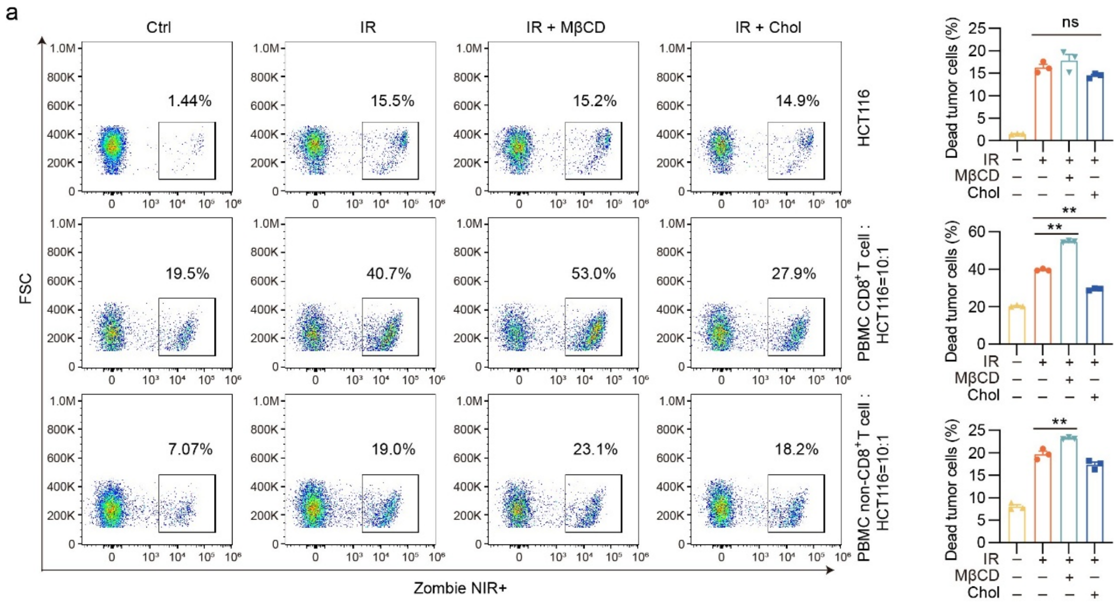

Supplementary Fig. 2 Cholesterol inhibits radiotherapy-mediated tumor cell killing by immune cells. (a) HCT116 cells with different treatments (ctrl, IR 6 Gy, IR + cholesterol 50 μM and IR + MβCD 2 mM) were co-cultured with CD8$^+$ T cells or non-CD8$^+$ T cells of PBMCs for a 36-hour period, where cholesterol was pre-treated for 12 hours and MβCD was pre-treated for 4 hours. The ratio of immune cells to tumor cells was 0:1 and 10:1, respectively, followed by flow cytometry analysis and quantitative analysis. $**P < 0.01$; ns: not significant.

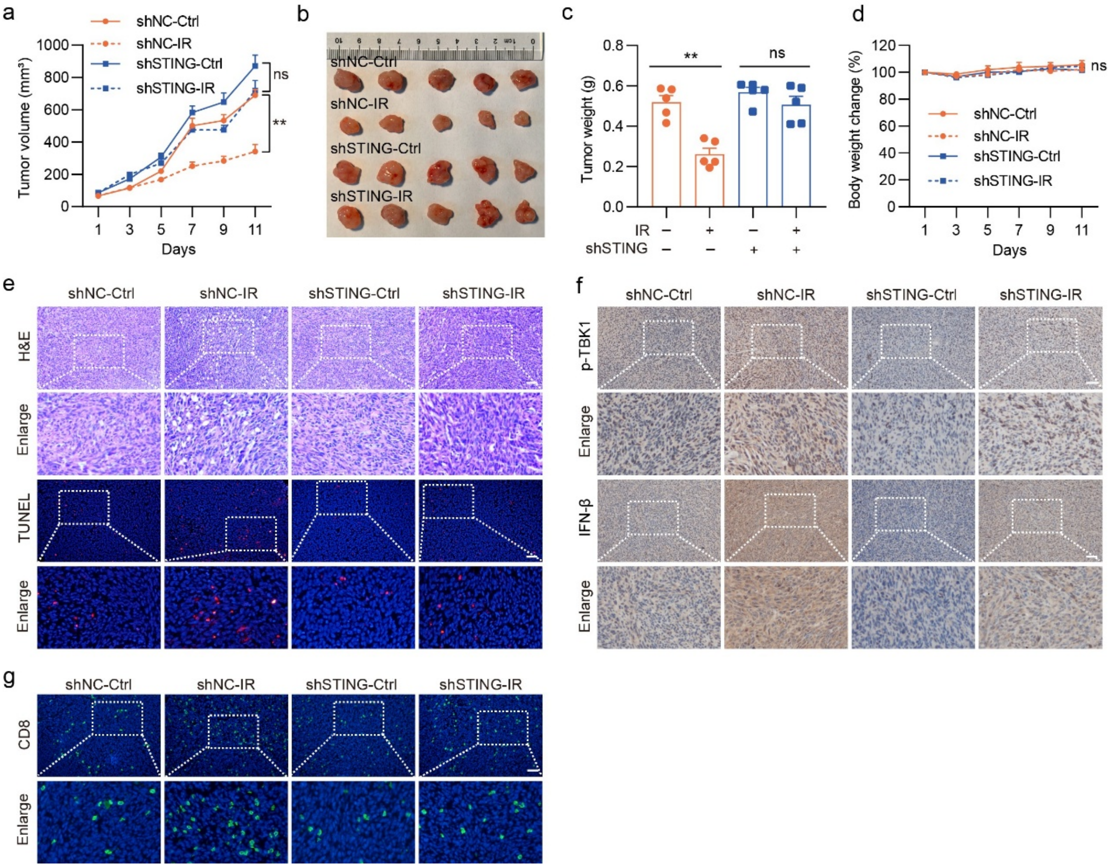

Supplementary Fig. 3 The presence of STING in tumor cells is a critical determinant of the efficacy of radiotherapy in eliciting anti-tumor immunity. (a) Tumor volume (mean ± SEM) was measured after radiotherapy (6 Gy) every two days. **(b)** Solid tumors were separated after the mice were sacrificed. **(c)** Tumor weight (mean ± SEM) was measured after the mice were sacrificed. **(d)** Body weight change (mean ± SEM) was measured after radiotherapy every two days. **(e)** The tumor tissue paraffin sections were subjected to H&E staining, and TUNEL staining. **(f)** Representative IHC images of p-TBK1 and IFNβ. **(g)** Representative IF images of tumor-infiltrating CD8^{+} T cells. Scale bar, 50 μm. ***P* < 0.01; ns: not significant.

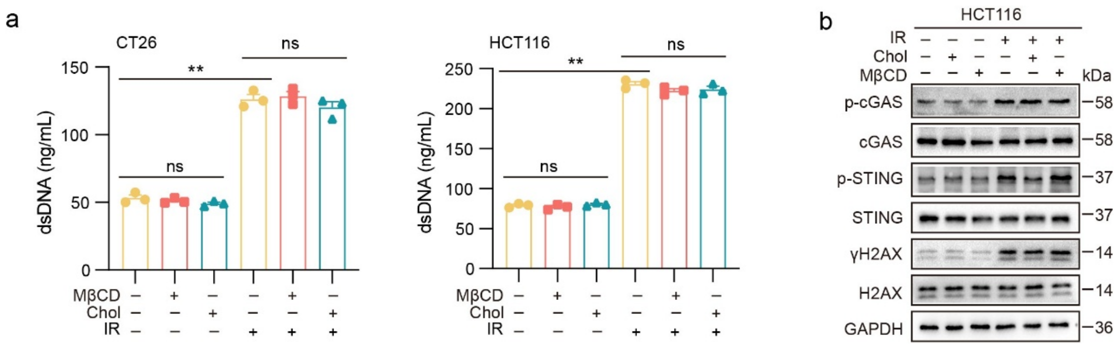

Supplementary Fig. 4 Cholesterol does not affect the activation of signaling pathways upstream of cGAS-STING signaling pathway. (a) The concentration of extracellular dsDNA in the culture supernatant of CT26 and HCT116 cells with different treatments (ctrl, cholesterol 50 μM, MβCD 2 mM, IR 6 Gy, IR + cholesterol, IR + MβCD). **(b)** The expression of p-cGAS, cGAS, p-STING, STING, γH2AX, and H2AX protein was extracted from HCT116 cells with different treatments and detected by western blot. ***P* < 0.01; ns: not significant.

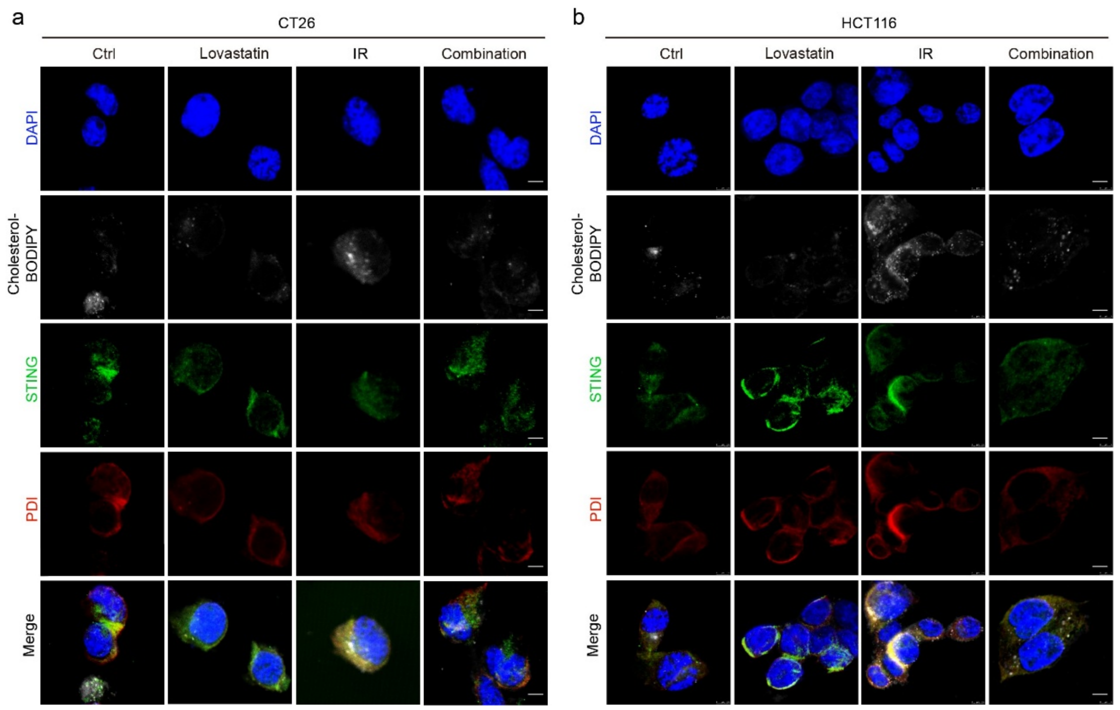

Supplementary Fig. 5 Cholesterol inhibits STING trafficking from ER to Golgi. (a, b) Co-localization analysis of cholesterol, STING and ER tracker (PDI) in different groups of CT26 (a) and HCT116 cells (b). Scale bar, 10 μm.

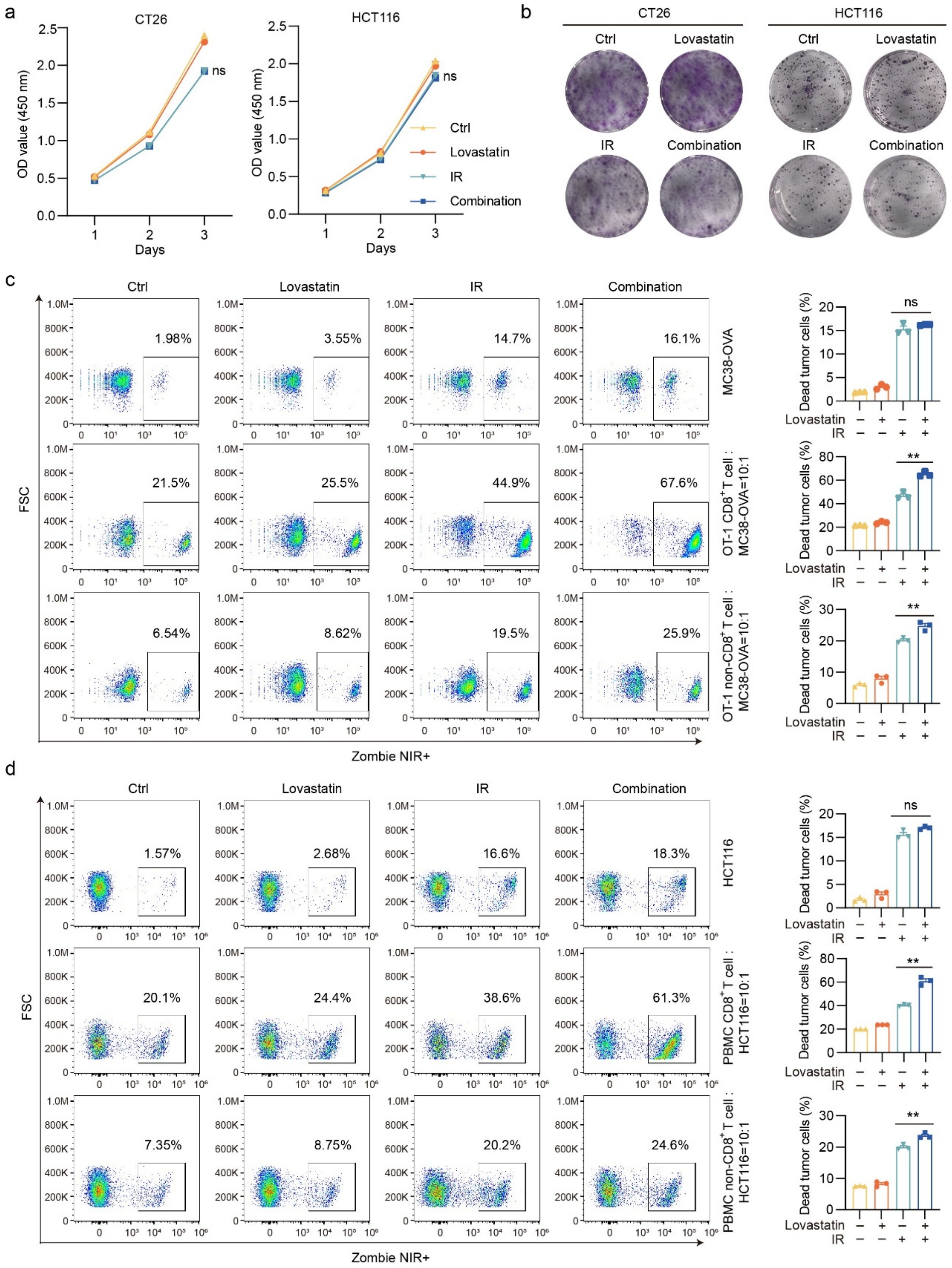

Supplementary Fig. 6 Lovastatin augments the efficacy of radiotherapy by fostering immune cell cytotoxicity in vitro. (a) CCK-8 assay was performed on CT26 and HCT116 cells with different treatments (ctrl, lovastatin 10 μM, IR 6 Gy and IR + lovastatin) over three consecutive days. **(b)** Clonogenic assay of CT26 and HCT116

cells with different treatments. **(c)** MC38-OVA cells with different treatments were co-cultured with $CD8^+$ T cells or non-$CD8^+$ T cells extracted from the spleen of OT-1 mice for a 36-hour period, and the ratio of immune cells to tumor cells is 0:1 and 10:1, followed by flow cytometry analysis and quantitative analysis. **(d)** HCT116 cells with different treatments were co-cultured with $CD8^+$ T cells or non-$CD8^+$ T cells of PBMCs for a 36-hour period, and the ratio of immune cells to tumor cells is 0:1 and 10:1, followed by flow cytometry analysis and quantitative analysis. $**P < 0.01$; ns: not significant.

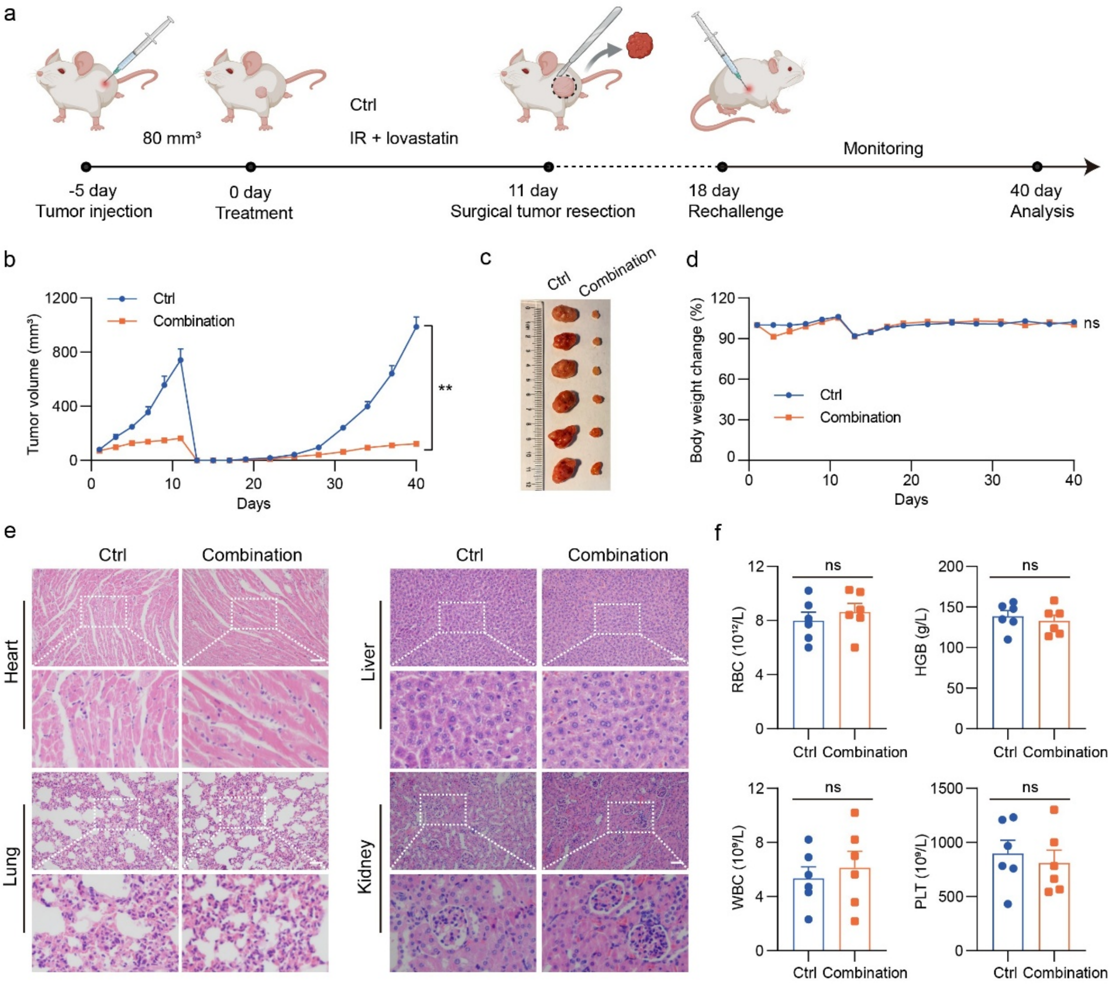

Supplementary Fig. 7 Lovastatin combined with radiotherapy treatment induces durable anti-tumor immunity in mice without increased toxicity. (a) Diagram of experimental design. **(b)** Tumor volume (mean ± SEM) was measured after radiotherapy every two or three days. **(c)** Solid tumors were separated after the

rechallenge mice were sacrificed. **(d)** Bodyweight change (mean ± SEM) was measured every two or three days. **(e)** H&E staining of major organs (heart, liver, lung, and kidney) of the rechallenge mice. **(f)** Variations of RBC (Red blood cell), HGB (Hemoglobin), WBC (White blood cell), and PLT (Platelet) in blood samples were analyzed. Scale bar, 50 μm. **P < 0.01; ns: not significant.

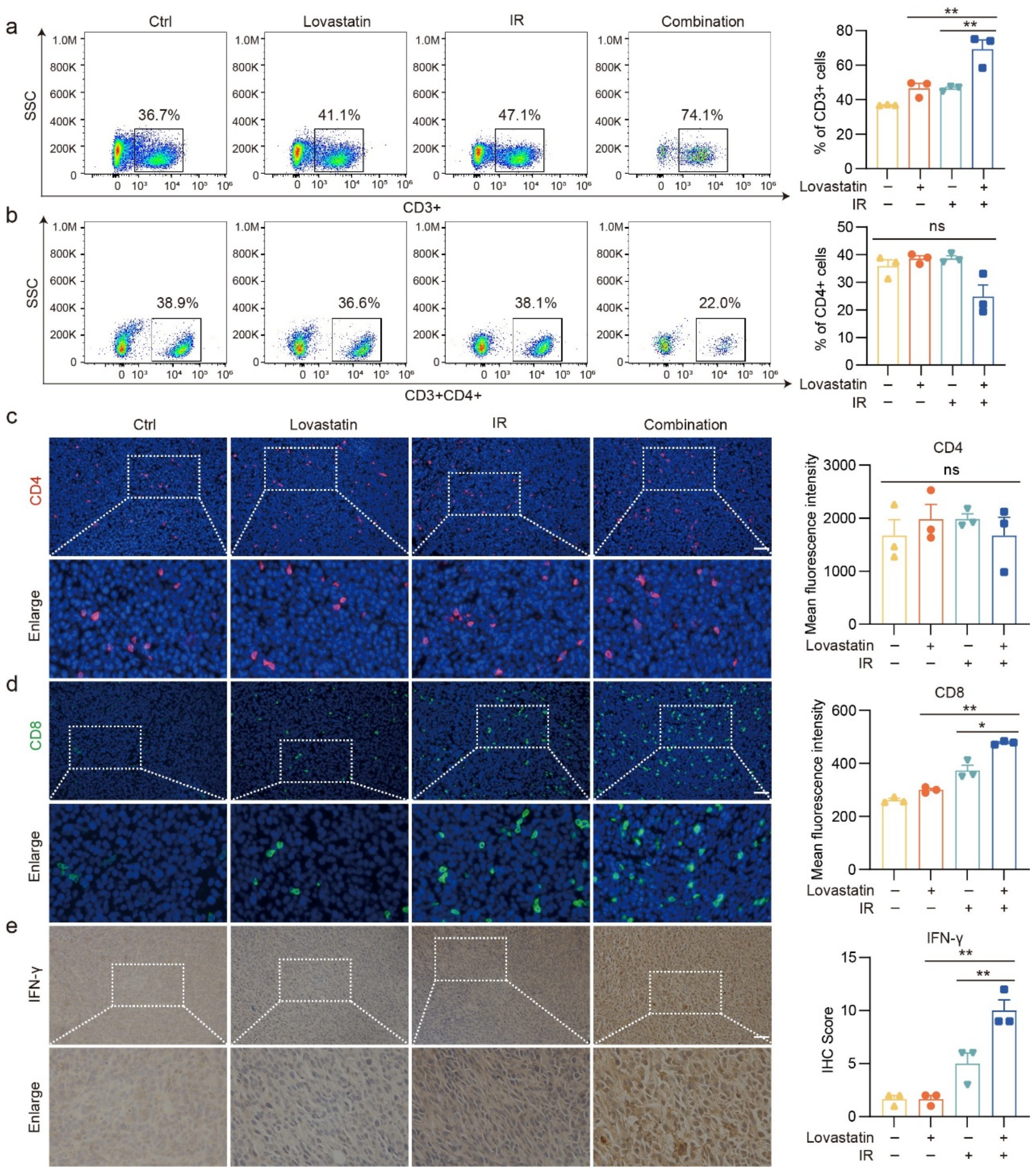

Supplementary Fig. 8 Lovastatin combined with radiotherapy enhances the infiltration and function of immune cells in CT26 tumor models. Flow cytometry,

IF staining and IHC staining were employed to assess tumor-infiltrating lymphocytes in the CT26 tumor models. **(a, b)** Examination of $CD3^+$ T, $CD3^+$ $CD4^+$ T cells in the tumor by flow cytometry and quantitative analysis. **(c, d)** Representative IF images and quantitative analysis of tumor-infiltrating $CD4^+$ T and $CD8^+$ T cells. **(e)** Representative IHC images and quantitative analysis of tumor-infiltrating IFN-γ. Scale bar, 50 μm. $^{**}P < 0.01$; $^{*}P < 0.05$; ns: not significant.
