# Supplementary material for: Cholesterol biosynthesis induced by radiotherapy inhibits cGAS–STING activation and contributes to colorectal cancer treatment resistance
Source: Exp Mol Med. 2025 May 12;57(5):1089–105. doi: 10.1038/s12276-025-01457-6 (PMC12130323; doi:10.1038/s12276-025-01457-6)
Supplement: Supplementary file 2 — Supplementary Tables 1 and 2 [file 12276_2025_1457_MOESM2_ESM.docx]

Table1 HCT116

| A | A*24:02:01; A*02:01:01 |
| --- | --- |
| B | B*08:01:01; B*46:01:01 |
| C | C*07:01:01; C*05:01:01 |
| DQB1 | DQB1*03:19:01; DQB1*02:01:01 |
| DPB1 | DPB1*104:01:01; DPB1*04:02:01 |
| DRB1 | DRB1*03:01:01; DRB1*11:02:01 |

Table2 Volunteer

| A | A*01:01:01; A*02:01:01 |
| --- | --- |
| B | B*45:01:01; B*18:01:01 |
| C | C*07:02:01; C*01:03:01 |
| DQB1 | DQB1*03:03:02; DQB1*02:01:01 |
| DPB1 | DPB1*05:01:01; DPB1*02:01:02 |
| DRB1 | DRB1*03:01:01; DRB1*09:01:02 |
